# Supplementary material for: Cancer cell sedimentation in 3D cultures reveals active migration regulated by self-generated gradients and adhesion sites
Source: PLoS Comput Biol. 2024 Jun 11;20(6):e1012112. doi: 10.1371/journal.pcbi.1012112 (PMC11195982; doi:10.1371/journal.pcbi.1012112)
Supplement: S1 File — (PDF) [file pcbi.1012112.s001.pdf]

Supporting Information: Cancer cell sedimentation in 3D cultures reveals active migration regulated by self-generated gradients and adhesion sites

Nikolaos M. Dimitriou[1*], Salvador Flores-Torres[1], Maria Kyriakidou[2], Joseph Matthew Kinsella[1], Georgios D. Mitsis[1*]

1 Department of Bioengineering, McGill University, Montreal, QC, Canada
2 Department of Human Genetics, McGill University, Montreal, QC, Canada

* nikolaos.dimitriou@mail.mcgill.ca, georgios.mitsis@mcgill.ca

Contents

1 Experiments **2**

2 Cell viability assay **3**

3 Continuum model calibration **8**

4 Normalized Root Mean Squared Error **8**

5 Model validation analysis **12**

List of Figures

A **Experiments.** Cell locations in the 3D space for (a) non-treatment (control), (b) 0.0005 μM, (c) 0.005 μM, (d) 0.05 μM, (e) 0.5 μM of Paclitaxel, respectively. (f) Number of segmented nuclei with respect to time. We observe that higher doses of Paclitaxel inhibit cell movement, while their number remains approximately the same across time, with a reduction occurring after the 9th day. 3

B **Experiments.** Histograms of numbers of cells with respect to the cell culture height. The comparisons are made between the non-treatment (control) and the Paclitaxel-treatment conditions. 4

C **RNA-seq data analysis pipeline.** The differential gene expression analysis was performed between non-treatment and Paclitaxel treatment conditions. 5

D **Gene Ontology (GO) over-representation test.** Among other ontologies, the Adherens Junction (AJ) is over-represented with adjust p-value ≈ 0.0015. 5

E **Cell migration mechanisms.** EMT signalling pathway did not follow a distinct pattern between treatment and non-treatment. As a result, the EMT mechanism was not considered as a plausible mechanism of migration. 6

F **Alternative possible mechanisms of migration.** Genes involved in a) sprouting and branching, b) contact inhibition of locomotion, c) supracellular contraction. C1 and C2 correspond to the control samples. The treatment samples are denoted with Pac following the dose of the treatment in μM. 6

G **Agarose coating experiment.** Cell viability was reduced compared to the control, non-coating experiment. Thus, surfaces of adhesion play a major part in cell viability in *in-vitro* conditions. 7

H **Hybrid Model** Cellular automaton (CA). (a) Flowchart of the CA. (b) Migration and spontaneous death probability parameters of the CA with respect to time. The migration of cells depends on the number of neighbours. The increase of the migration parameter with respect to time is based on observations made on the experiments showing that cells tended to form clusters during the later time-points. The spontaneous death probability was measured using a flow cytometry experiment. 7

I **Continuum model calibration.** Posterior distributions of the model parameters for representative datasets of (a) non-treatment conditions, (b) 0.0005 µM, (c) 0.005 µM, (d) 0.05 µM, (e) 0.5 µM of Paclitaxel. Above diagonal: Projected TMCMC samples of the posterior distribution in 2D space. Diagonal: Marginals of the joint posterior obtained via kernel densities. Red lines denote the 95% credible intervals. Below diagonal: 2D projected densities of the posterior obtained using 2D kernel densities. 10
J **Continuum model calibration.** Contour plots for the y-z cross-section at x = 1.25 mm. a)-d) No treatment, e)-h) 0.0005 µM of Paclitaxel, i)-l) 0.005 µM of Paclitaxel, m)-p) 0.05 µM of Paclitaxel, q)-t) 0.5 µM of Paclitaxel for days 7, 9, 12, 14. Red: experiments, Black: simulations. 11
K **Model validation analysis.** Clustering abundance for (a) Experiments, (b) Simulations with uniform death probability across the space, and (c) Simulations with a radial decrease in cell death probability towards the center of the space. 13
L **Spatial analysis.** Inter-Nucleic Euclidean distance distributions between (a) experiments, and (b) simulations across time. Overall, the distributions remain stable across time and treatment condition in both experiments and simulations. 13
M **Spatial analysis.** Nearest-Neighbour Euclidean distance distributions of the non-treatment conditions across time. The NN distances initially formed wide distributions that gradually tended to become narrower around lower neighbourhood radii values with respect to time, across all samples, with similar characteristic peaks at ∼15 µm. 14
N **Spatial analysis.** Relationship between diffusion and advection mechanisms with morphological patterns. The K-function was calculated for 12 simulations of two cases; one with low advection (0.0005 ± 0.0004 mm^2/day) and high diffusion (0.0076 ± 0.0003 mm^2/day) constants, and one with high advection (0.0052 ± 0.0002 mm^2/day) and low diffusion (0.00186 ± 0.00009 mm^2/day) constants. The area under the resulted K-function (AUC$_K$) was then calculated, and it was found that the combination low advection and high diffusion constants produced a considerably lower AUC$_K$ than the combination of high advection and low diffusion constants. The statistical significance of this result was calculated using the Kruskal-Wallis test, and it was found that p-value$\approx 5.4 \times 10^{-5}$. 14

List of Tables

A **Continuum model calibration** Average and standard deviation of the inferred parameter values of the continuum KS model across all datasets of the non-treatment condition (control). The prior PDF boundaries used for the parameter estimation are shown in the header of the table. 9
B **Continuum model calibration** Average and standard deviation of the inferred parameter values of the continuum KS model across all datasets of the Paclitaxel treatment with concentration 0.0005 µM. The prior PDF boundaries used for the parameter estimation are shown in the header of the table. 9
C **Continuum model calibration** Average and standard deviation of the inferred parameter values of the continuum KS model across all datasets of the Paclitaxel treatment with concentration 0.005 µM. The prior PDF boundaries used for the parameter estimation are shown in the header of the table. 9
D **Continuum model calibration** Average and standard deviation of the inferred parameter values of the continuum KS model across all datasets of the Paclitaxel treatment with concentration 0.05 µM. The prior PDF boundaries used for the parameter estimation are shown in the header of the table. 9
E **Continuum model calibration** Average and standard deviation of the inferred parameter values of the continuum KS model across all datasets of the Paclitaxel treatment with concentration 0.5 µM. The prior PDF boundaries used for the parameter estimation are shown in the header of the table. 10

1 Experiments

Overall, we performed 5 different experiments that enabled us understand the behaviour of TNBC cells of the MDA-MB-231 cell line in 3D cultures; (i) a simple 3D culture experiment (control) where the cells migrated towards the glass bottom of the plate, (ii) a 3D culture experiment with migration inhibition using the drug Paclitaxel, (iii) a 3D culture for

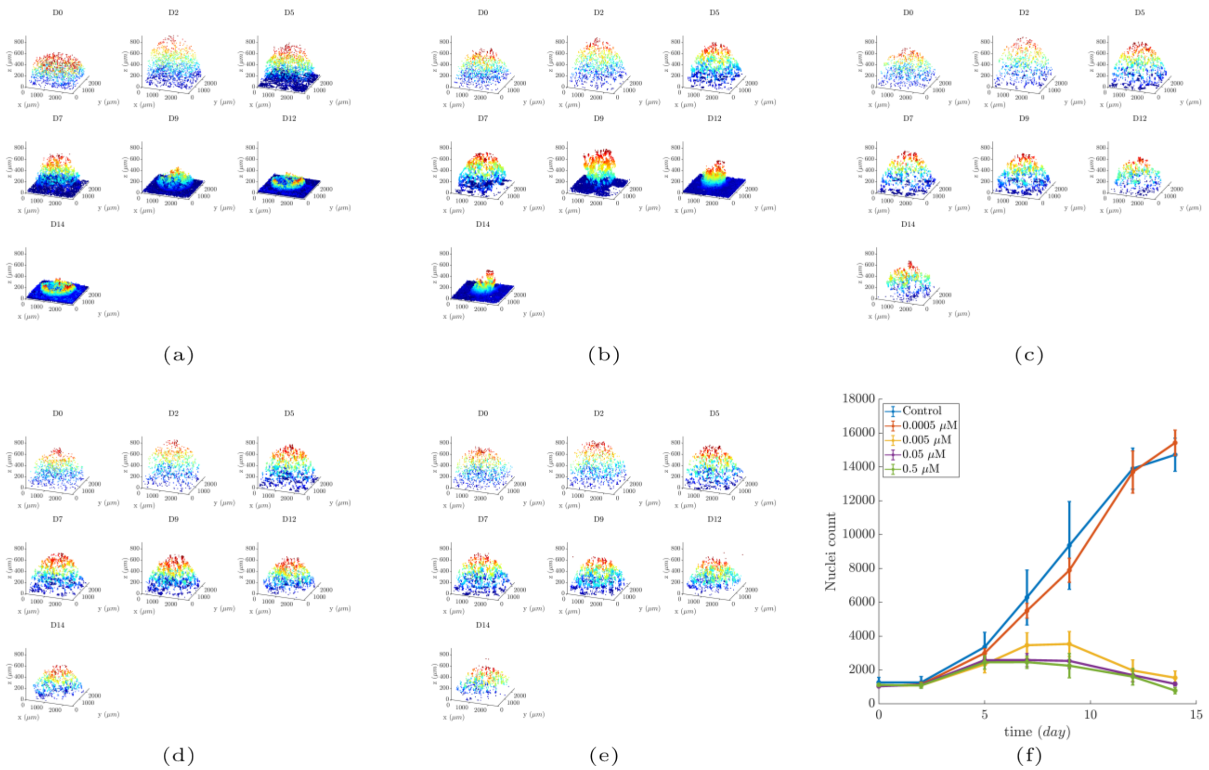

Fig A Experiments. Cell locations in the 3D space for (a) non-treatment (control), (b) 0.0005 µM, (c) 0.005 µM, (d) 0.05 µM, (e) 0.5 µM of Paclitaxel, respectively. (f) Number of segmented nuclei with respect to time. We observe that higher doses of Paclitaxel inhibit cell movement, while their number remains approximately the same across time, with a reduction occurring after the 9th day.

RNA-sequencing experiment under Control (non-treatment), and Paclitaxel treatment conditions, (iv) a 3D culture experiment that included agarose coating of the glass bottom, and (v) a 3D co-culture experiment with the TNBC cells, and fibroblasts.

As described in the manuscript, sedimentation was inhibited in presence of the drug Paclitaxel, and specifically for concentrations 0.5 µM, 0.05 µM, and 0.005 µM. This is further depicted in Figs A, B. Further analysis of bulk RNA-seq data between non-treatment and Paclitaxel-treatment conditions revealed active migration mechanisms upregulated in the non-treatment data. For the RNA-seq experiment the analysis pipeline is presented in Fig C. The Gene Ontology over-representation test (Fig D) revealed that many of the over-represented ontologies link to cellular mechanisms of migration. Particularly the Adherens Junction (AJ) signalling together with MAPK, and TGF-β are shown to contribute to a collective mode of migration, as described in [1]. A further search on the scenario of a possible individual migration mechanism regulated by EMT, we found that genes involved in the EMT migration mechanism have no distinct pattern of expression between non-treatment and treatment conditions (Fig E). Hence, migration via the EMT mechanism was less likely to occur compared to collective migration via MAPK, TGF-β, and AJ signalling. Additionally, we examined other possible mechanisms of migration whose expression patterns are found in Fig F.

For the 3D culture experiment that included agarose coating of the glass bottom, as described in the manuscript, the cells in the coated sample stopped moving towards the bottom, indicating the important role of the glass in cell migration. Additionally, we discussed that the viability of the cells was reduced in the coated samples, as shown in Fig G.

2 Cell viability assay

To estimate the viability of the cells during the plateau phase of the logistic curve, we performed a cell viability test on the 3D cultures using flow cytometry. The number of dead cells was measured for days 11, 12, and 13 after the time of seeding.

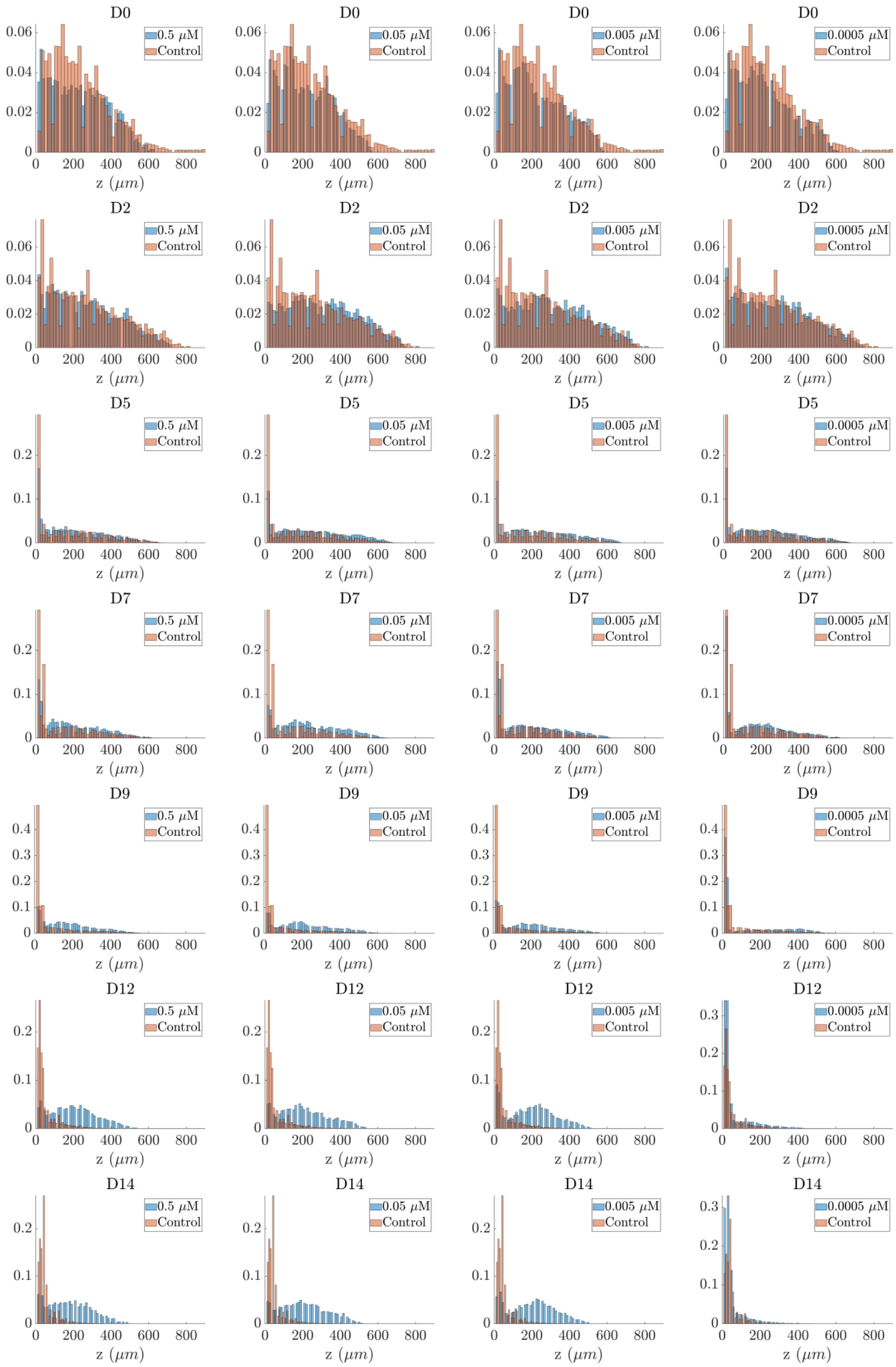

Fig B Experiments. Histograms of numbers of cells with respect to the cell culture height. The comparisons are made between the non-treatment (control) and the Paclitaxel-treatment conditions.

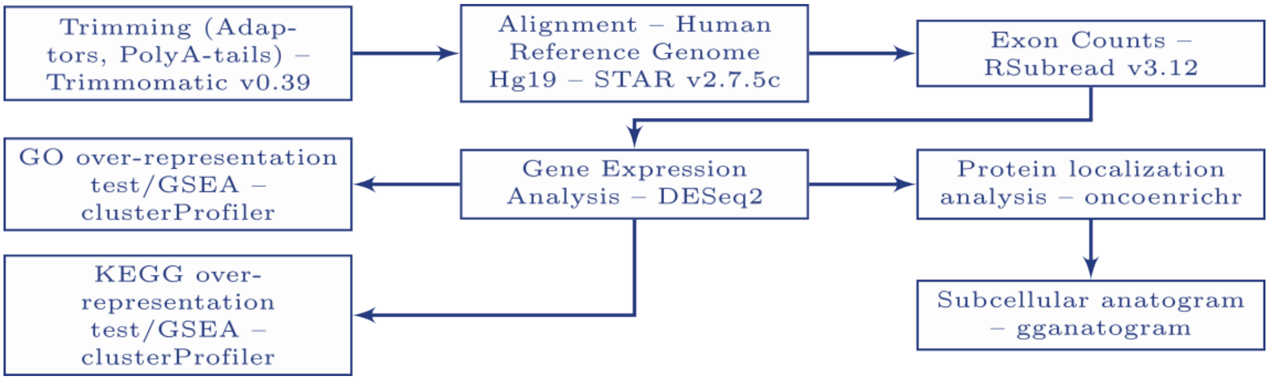

Fig C RNA-seq data analysis pipeline. The differential gene expression analysis was performed between non-treatment and Paclitaxel treatment conditions.

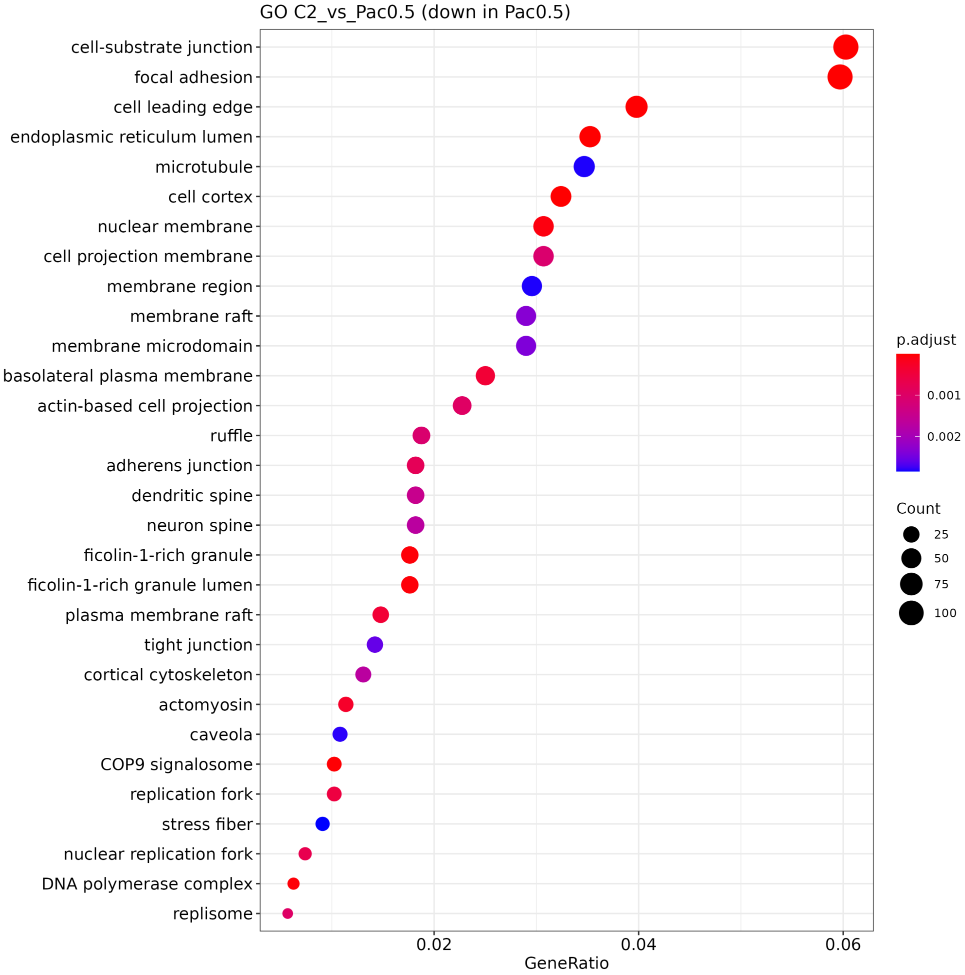

Fig D Gene Ontology (GO) over-representation test. Among other ontologies, the Adherens Junction (AJ) is over-represented with adjust p-value ≈ 0.0015.

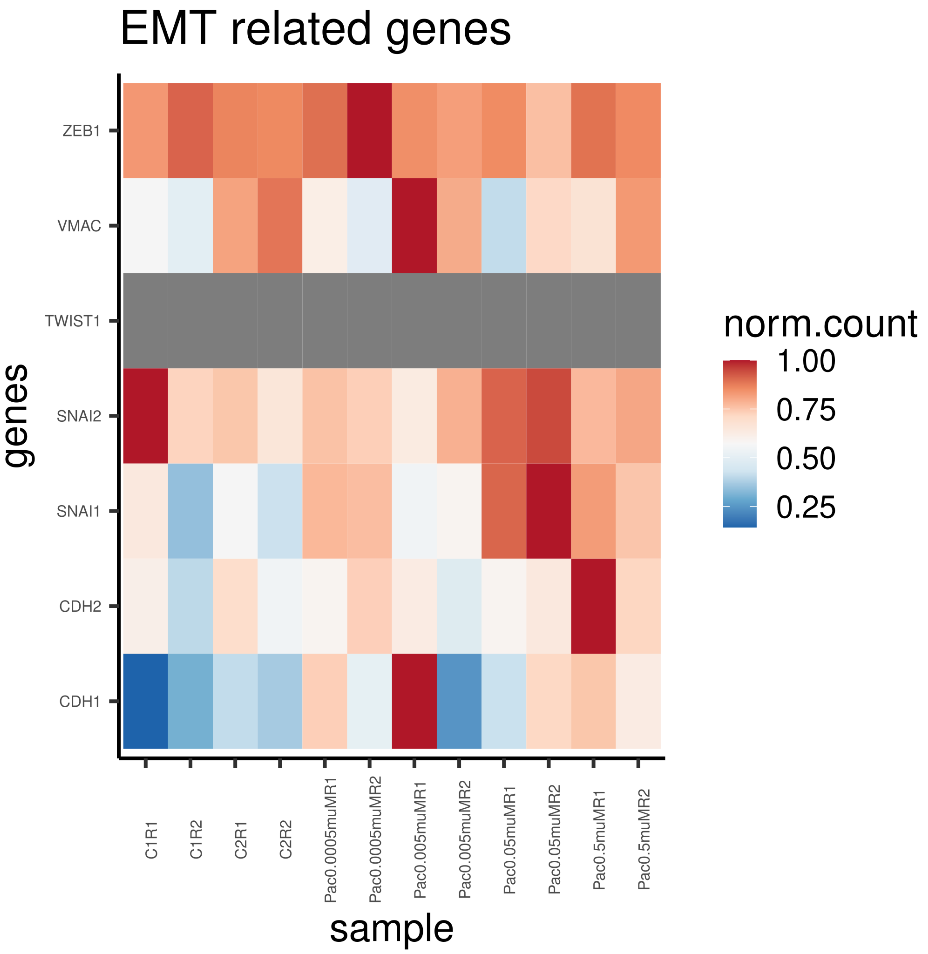

Fig E Cell migration mechanisms. EMT signalling pathway did not follow a distinct pattern between treatment and non-treatment. As a result, the EMT mechanism was not considered as a plausible mechanism of migration.

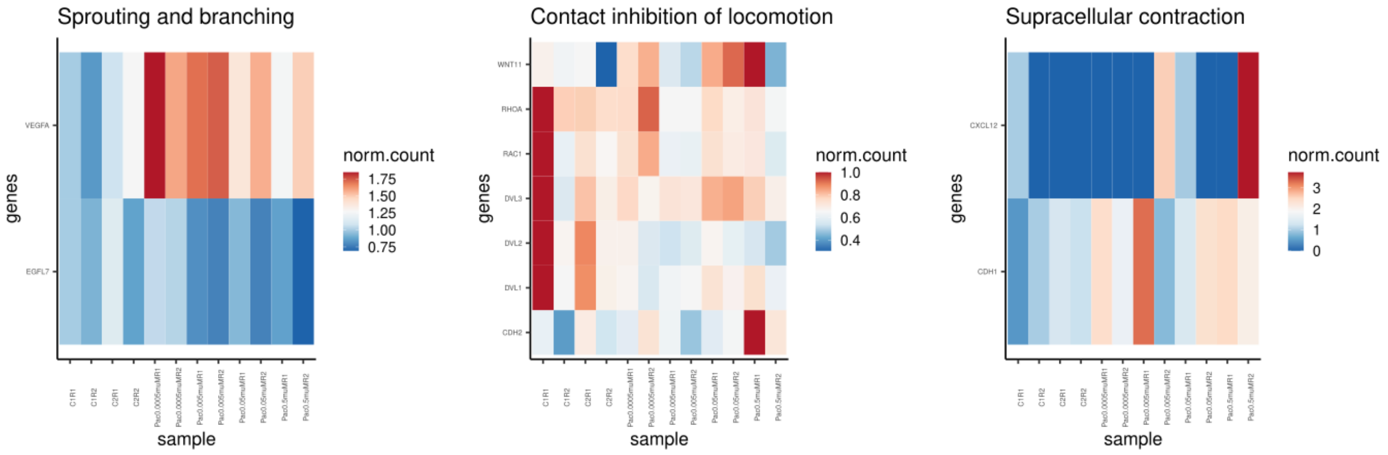

(a) (b) (c)

Fig F Alternative possible mechanisms of migration. Genes involved in a) sprouting and branching, b) contact inhibition of locomotion, c) supracellular contraction. C1 and C2 correspond to the control samples. The treatment samples are denoted with Pac following the dose of the treatment in μM.

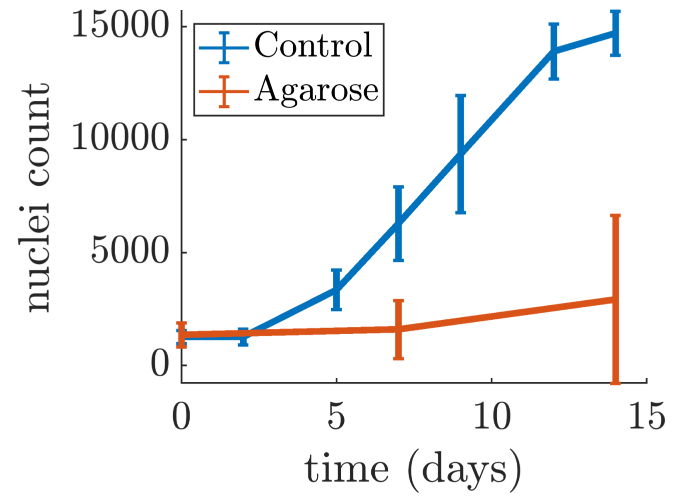

Fig G Agarose coating experiment. Cell viability was reduced compared to the control, non-coating experiment. Thus, surfaces of adhesion play a major part in cell viability in *in-vitro* conditions.

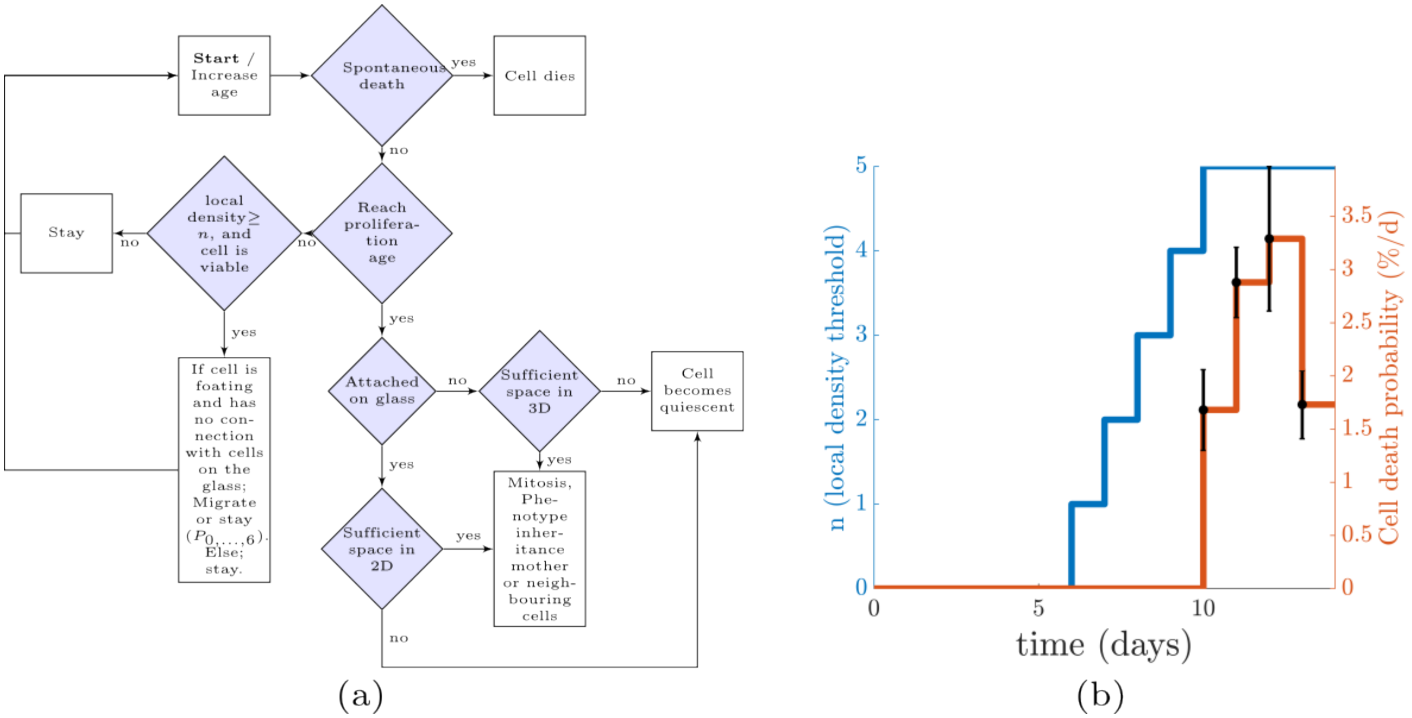

Fig H Hybrid Model Cellular automaton (CA). (a) Flowchart of the CA. (b) Migration and spontaneous death probability parameters of the CA with respect to time. The migration of cells depends on the number of neighbours. The increase of the migration parameter with respect to time is based on observations made on the experiments showing that cells tended to form clusters during the later time-points. The spontaneous death probability was measured using a flow cytometry experiment.

For each sample, the following protocol was implemented. Time 0 was acquired from non-encapsulated cells and every other time point corresponds to cells extracted from Matrigel domes. Fresh cell culture media was warmed at 37°C and supplemented with collagenase and dispase 1X (Sigma Aldrich, Cat. # 11097113001). Micropipettes were used to break down the cell laden Matrigel constructs by suction force, and these were incubated for 30 minutes inside a cell culture incubator (95% relative humidity, 37°C, and 5% CO2). Cells were centrifuged at 500g for 5 minutes, the supernatant was discarded and a 0.05% trypsin (Wisent, Cat. # 325-042-CL) was used to break any cell-to-cell adhesions for a period of 5 min. Trypsin was neutralized with cell culture medium, and the cell pellet was recollected after 5 min of centrifugation at 500g. A solution containing warm (37°C) 1X DPBS and 1X propidium iodine (miltenyi biotech, Cat. # 130-093-233) was used to resuspend and incubate the collected cells for 15 minutes. Finally, cells were centrifuged again at 500g for 5 minutes, the supernatant was discarded and they were resuspended in DPBS prior to flow cytometry experimentation. All time-points were acquired in triplicates and a PI+ control was implemented using fixed (4% PFA, 5 min) and pierced cells (0.01% SDS, 1 min). Dead cells were identified using the gating strategy described in Crowley et al. [2]. The percentage of dead cells was used to directly obtain the cell death probability rate, which is presented in Fig Hb of the manuscript.

3 Continuum model calibration

The parameters of the continuum were estimated using Baysian inference and Transitional Markov Chain Monte Carlo algorithm (TMCMC) [3] that is implemented in the Pi4U package [4]. The TMCMC algorithm estimated posterior distributions of the model parameters given the experimental data. The process was repeated for each dataset separately across the non-treatment and treatment conditions. In total, 32 estimations were performed, and each of them evaluated 14000-20000 sets of model parameters.

The TMCMC algorithm implemented a series of resampling stages. Initially, samples from a uniform prior distribution were drawn. Then, in each stage, samples of the model parameters are drawn with respect to PDF of the corresponding stage. The plausibility weights between the proposed samples and the sample of the previous stage are computed, as shown in Eq (1). The algorithm resampled based on the normalized weights (Eq (2)). At the last stage, the algorithm generated samples based on the previous stage, that are distributed as in Eq (3).

$$w(\underline{\theta}_{j,k}) = \frac{p_{j+1}(\underline{\theta}_{j,k})}{p_j(\underline{\theta}_{j,k})} = \frac{p(D|\underline{\theta}_{j,k}, M)^{\rho_{j+1}} p(\underline{\theta}_{j,k}|M)}{p(D|\underline{\theta}_{j,k}, M)^{\rho_j} p(\underline{\theta}_{j,k}|M)} = p(D|\underline{\theta}_{j,k}, M)^{\rho_{j+1}-\rho_j} \tag{1}$$

$$\underline{\tilde{w}}_{j,l} = \frac{w(\underline{\theta}_{j,l})}{\sum_{l=1}^{N_j} w(\underline{\theta}_{j,l})} \tag{2}$$

$$p(\underline{\theta}) \sim p(D|\underline{\theta}, M)p(\underline{\theta}|M) \tag{3}$$

The summary of the average and standard deviation of the parameter values in the posterior distributions across the examined conditions are found in Tables A-E. The posterior distributions of the model parameters for representative datasets across each condition are depicted in Fig I.

4 Normalized Root Mean Squared Error

The differences between the *in-vitro* and *in-silico* estimated cell density profiles were calculated using the Normalized Root Mean Squared Error (NRMSE) that is defined in Eq (4), where $u_e,\ u_s$ are the arrays containing the experimental and simulated density profiles, respectively.

$$\text{NRMSE} = \frac{1}{\max(u_e) - \min(u_e)} \sqrt{\frac{\sum_{i=1}^{N} (u_s^i - u_e^i)^2}{N}} \tag{4}$$

Dataset	$D_u \in [10^{-3}, 2] \times 10^{-3}$ (mm^2d^{-1})	$s \in [1.8, 3] \times 10^{-1}(d^{-1})$	$\chi \in [0.8, 5] \times 10^{-2}(mm^2d^{-1})$	$D_f \in [10^{-3}, 2] \times 10^{-3}$ (mm^2d^{-1})
1	1.999 ± 0.001	1.843 ± 0.001	0.833 ± 0.001	1.992 ± 0.003
2	1.698 ± 0.138	2.164 ± 0.140	0.819 ± 0.014	1.792 ± 0.120
3	1.872 ± 0.070	1.805 ± 0.003	0.883 ± 0.028	1.724 ± 0.149
4	0.006 ± 0.001	1.841 ± 0.017	0.830 ± 0.028	1.404 ± 0.050
5	1.995 ± 0.004	1.806 ± 0.005	0.802 ± 0.001	1.983 ± 0.006
6	1.807 ± 0.001	2.463 ± 0.001	0.936 ± 0.001	1.229 ± 0.001
7	0.205 ± 0.014	1.803 ± 0.002	1.063 ± 0.068	0.895 ± 0.108
8	0.328 ± 0.026	1.998 ± 0.016	2.050 ± 0.069	1.748 ± 0.019
9	0.244 ± 0.022	1.823 ± 0.011	2.414 ± 0.166	1.703 ± 0.124
10	1.854 ± 0.085	2.243 ± 0.020	0.803 ± 0.002	0.118 ± 0.019
11	1.951 ± 0.035	2.350 ± 0.073	0.804 ± 0.003	1.840 ± 0.034
12	1.514 ± 0.090	1.871 ± 0.036	0.874 ± 0.024	0.032 ± 0.003

Table A Continuum model calibration Average and standard deviation of the inferred parameter values of the continuum KS model across all datasets of the non-treatment condition (control). The prior PDF boundaries used for the parameter estimation are shown in the header of the table.

Dataset	$D_u \in [0, 2] \times 10^{-3}$ (mm^2d^{-1})	$s \in [1.92, 2.08] \times 10^{-1}(d^{-1})$	$k \in [0.0, 0.2](d^{-1})$	$\chi \in [0, 5] \times 10^{-2}(mm^2d^{-1})$	$D_f \in [1.2, 1.5] \times 10^{-3}$ (mm^2d^{-1})
1	1.5510 ± 0.0689	2.0417 ± 0.0041	0.0032 ± 0.0016	1.0475 ± 0.0188	1.2866 ± 0.0083
2	1.2040 ± 0.0088	2.0350 ± 0.0007	0.0506 ± 0.0074	0.0106 ± 0.0053	1.2418 ± 0.0003
3	1.7316 ± 0.0246	2.0810 ± 0.0032	0.0028 ± 0.0029	1.1428 ± 0.0096	1.4006 ± 0.0116
4	1.1329 ± 0.0005	2.0840 ± 0.0000	0.0100 ± 0.0003	2.0913 ± 0.0004	1.2693 ± 0.0001
5	1.7862 ± 0.1308	2.0779 ± 0.0021	0.0520 ± 0.0092	1.9857 ± 0.0672	1.2636 ± 0.0043

Table B Continuum model calibration Average and standard deviation of the inferred parameter values of the continuum KS model across all datasets of the Paclitaxel treatment with concentration 0.0005 µM. The prior PDF boundaries used for the parameter estimation are shown in the header of the table.

Dataset	$D_u \in [0.0, 10^{-2}] \times 10^{-2}$ (mm^2d^{-1})	$s \in [1.92, 2.08] \times 10^{-1}(d^{-1})$	$k \in [2, 5] \times 10^{-1}(d^{-1})$	$\chi \in [0.0, 10^{-3}] \times 10^{-2}(mm^2d^{-1})$	$D_f \in [1.2, 1.5] \times 10^{-3}$ (mm^2d^{-1})
1	0.0553 ± 0.0049	2.0638 ± 0.0083	2.0081 ± 0.0056	0.0004 ± 0.0001	1.2559 ± 0.0120
2	0.0675 ± 0.0017	2.0822 ± 0.0008	2.0009 ± 0.0006	0.0004 ± 0.0001	1.2439 ± 0.0029
3	0.0755 ± 0.0041	2.0822 ± 0.0010	2.0005 ± 0.0005	0.0005 ± 0.0001	1.2719 ± 0.0068
4	0.0688 ± 0.0011	2.0826 ± 0.0008	2.0015 ± 0.0010	0.0004 ± 0.0001	1.2448 ± 0.0023
5	0.0757 ± 0.0053	2.0801 ± 0.0030	2.0028 ± 0.0028	0.0003 ± 0.0001	1.3129 ± 0.0114

Table C Continuum model calibration Average and standard deviation of the inferred parameter values of the continuum KS model across all datasets of the Paclitaxel treatment with concentration 0.005 µM. The prior PDF boundaries used for the parameter estimation are shown in the header of the table.

Dataset	$D_u \in [0.0, 10^{-2}] \times 10^{-3}$ (mm^2d^{-1})	$s \in [1.92, 2.08] \times 10^{-1}(d^{-1})$	$k \in [2, 5] \times 10^{-1}(d^{-1})$	$\chi \in [0.0, 10^{-3}] \times 10^{-2}(mm^2d^{-1})$	$D_f \in [1.2, 1.5] \times 10^{-3}$ (mm^2d^{-1})
1	0.0067 ± 0.0001	2.0836 ± 0.0003	2.0009 ± 0.0006	0.0005 ± 0.0001	1.2560 ± 0.0054
2	0.0054 ± 0.0003	2.0810 ± 0.0013	2.0023 ± 0.0016	0.0004 ± 0.0001	1.2745 ± 0.0086
3	0.0072 ± 0.0004	2.0509 ± 0.0078	2.0105 ± 0.0091	0.0002 ± 0.0001	1.3234 ± 0.0254
4	0.0072 ± 0.0007	2.0803 ± 0.0026	2.0022 ± 0.0018	0.0006 ± 0.0001	1.3097 ± 0.0303
5	0.0053 ± 0.0006	2.0768 ± 0.0061	2.0054 ± 0.0053	0.0007 ± 0.0002	1.3945 ± 0.0460

Table D Continuum model calibration Average and standard deviation of the inferred parameter values of the continuum KS model across all datasets of the Paclitaxel treatment with concentration 0.05 µM. The prior PDF boundaries used for the parameter estimation are shown in the header of the table.

Dataset	$D_u \in [0.0, 10^{-2}] \times 10^{-3}$ $(mm^2 d^{-1})$	$s \in [1.92, 2.08] \times 10^{-1}(d^{-1})$	$k \in [2, 5] \times 10^{-1}(d^{-1})$	$\chi \in [0.0, 10^{-3}] \times 10^{-2}(mm^2 d^{-1})$	$D_f \in [1.2, 1.5] \times 10^{-3}$ $(mm^2 d^{-1})$
1	0.0097 ± 0.0003	2.0030 ± 0.0127	2.5438 ± 0.0211	0.0003 ± 0.0001	1.2738 ± 0.0261
2	0.0081 ± 0.0001	2.0836 ± 0.0003	2.5327 ± 0.0137	0.0001 ± 0.0001	1.2734 ± 0.0041
3	0.0054 ± 0.0003	2.0815 ± 0.0012	2.0015 ± 0.0013	0.0007 ± 0.0001	1.3193 ± 0.0189
4	0.0067 ± 0.0007	2.0682 ± 0.0068	2.0078 ± 0.0066	0.0005 ± 0.0001	1.3828 ± 0.0221
5	0.0072 ± 0.0007	2.0701 ± 0.0056	2.0070 ± 0.0053	0.0004 ± 0.0001	1.2670 ± 0.0212

Table E Continuum model calibration Average and standard deviation of the inferred parameter values of the continuum KS model across all datasets of the Paclitaxel treatment with concentration 0.5 µM. The prior PDF boundaries used for the parameter estimation are shown in the header of the table.

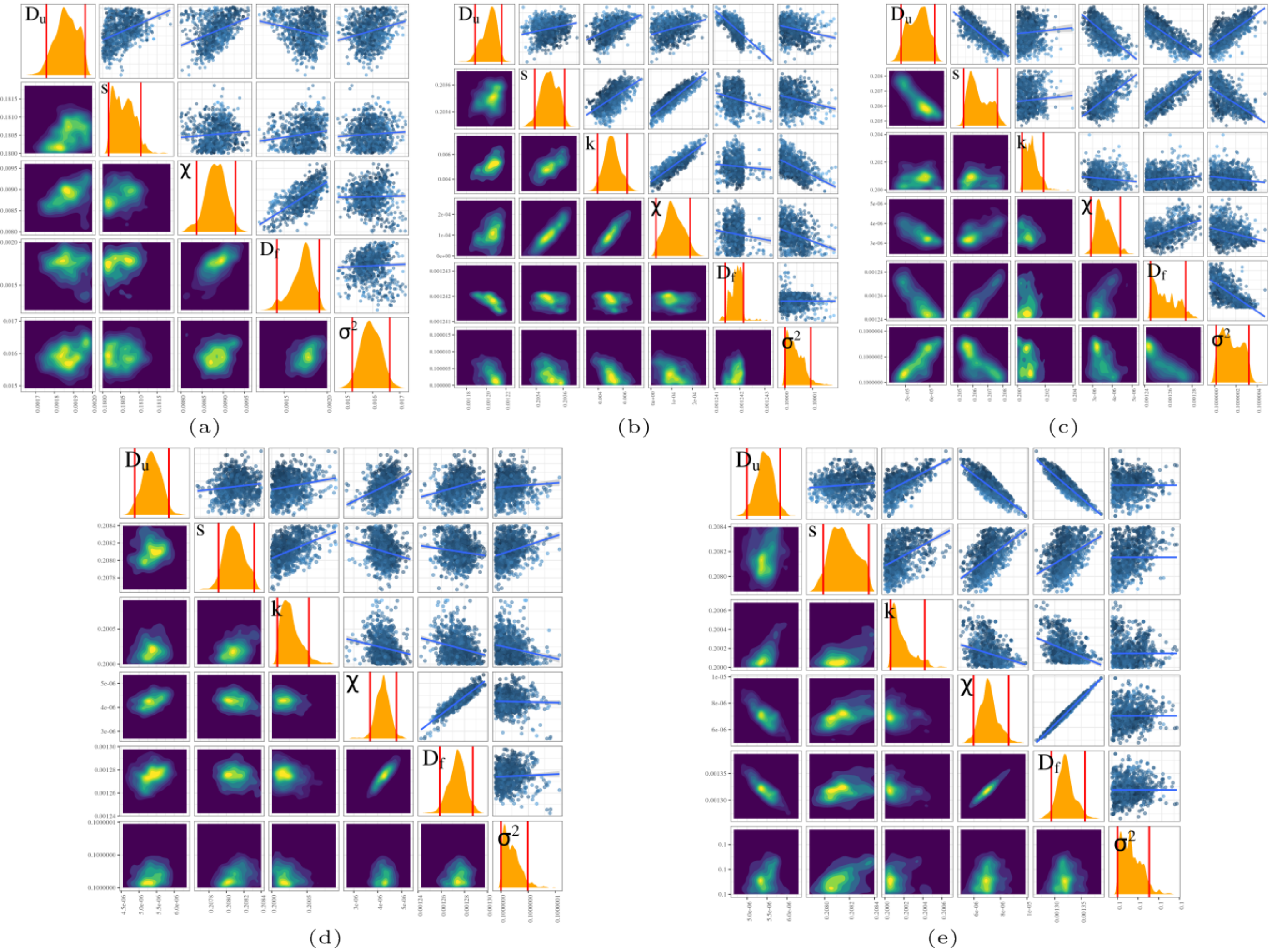

Fig I Continuum model calibration. Posterior distributions of the model parameters for representative datasets of (a) non-treatment conditions, (b) 0.0005 µM, (c) 0.005 µM, (d) 0.05 µM, (e) 0.5 µM of Paclitaxel. Above diagonal: Projected TMCMC samples of the posterior distribution in 2D space. Diagonal: Marginals of the joint posterior obtained via kernel densities. Red lines denote the 95% credible intervals. Below diagonal: 2D projected densities of the posterior obtained using 2D kernel densities.

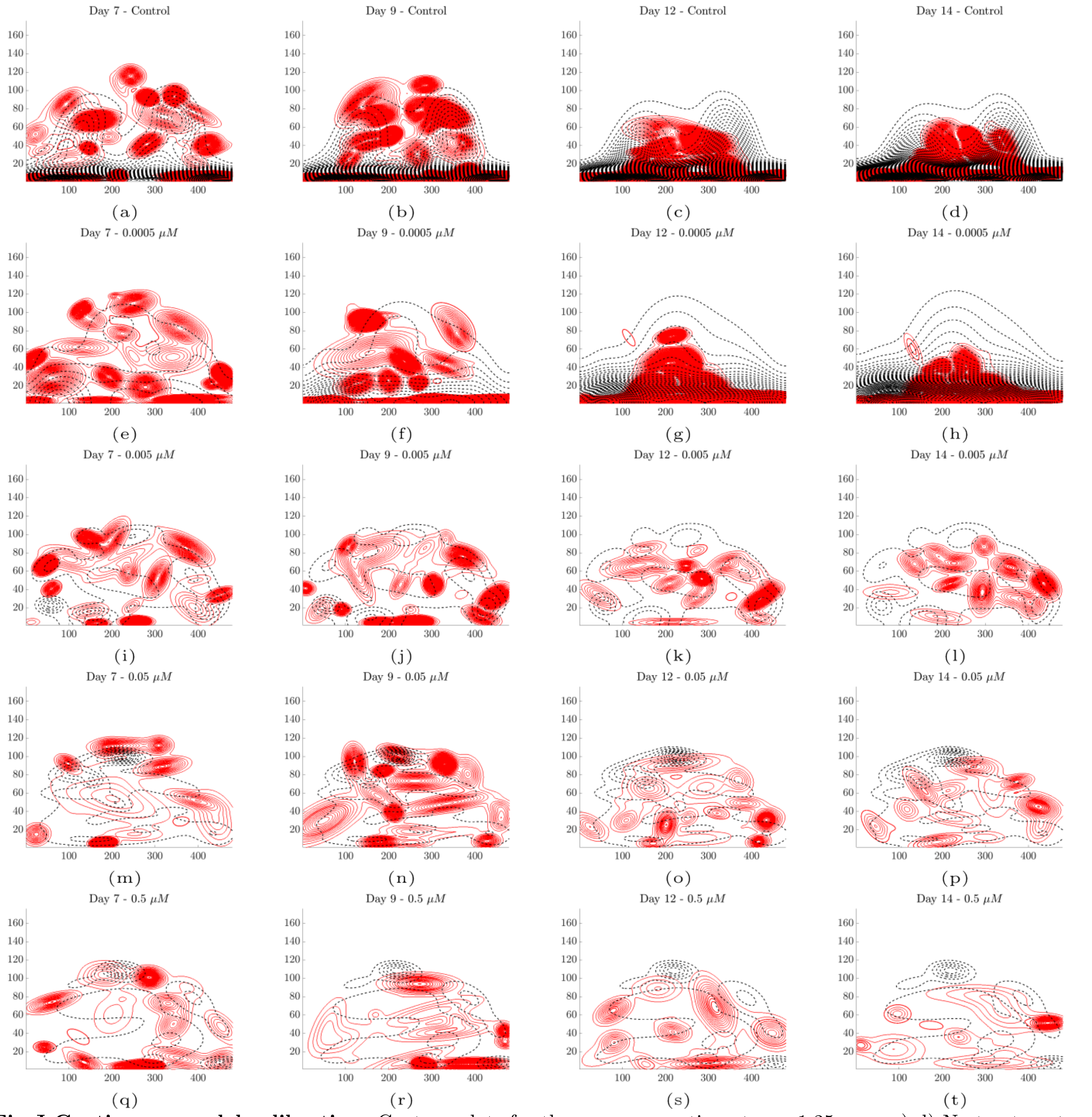

Fig J Continuum model calibration. Contour plots for the y-z cross-section at x = 1.25 mm. a)-d) No treatment, e)-h) 0.0005 µM of Paclitaxel, i)-l) 0.005 µM of Paclitaxel, m)-p) 0.05 µM of Paclitaxel, q)-t) 0.5 µM of Paclitaxel for days 7, 9, 12, 14. Red: experiments, Black: simulations.

5 Model validation analysis

For the analysis of model validity, we performed the Complete Spatial Randomness test using Ripley's K-function, and we calculated the Nearest-Neighbour, and the Inter-Nucleic Euclidean distance distributions. The results of these tests were compared with the results obtained from the experimental data and overall they exhibited a good agreement. In this section we present the results obtain from the calculation of the Inter-Nucleic and Nearest-Neighbour Euclidean distances between the cells (Figs L, M). The distributions remain stable across time, and condition indicating that the behaviour of cells is the same across the examined space. In contrast, the Nearest-Neighbour distances became narrower over time with characteristic peaks at 15µm.

The CSR test revealed pronounced clustered patterns across all datasets, with the experimental data exhibiting increasing clustering with respect to time and dose increment. The amount of clustering was quantified by calculating the area under the curve of the K-function (AUC_K), and the normalized AUC_K that is $\hat{\mathrm{AUC}}_{K,i} = \mathrm{AUC}_{K,i}/\max(\mathrm{AUC}_K)$ is presented in Fig Ka. In contrast, the simulation results showed decreasing clustering with respect to dose increase (Fig Kb). We attributed this result to the fact that the *in-silico* cells have uniform probability of dying across the space, while in the experiment, the drug concentration may have formed a gradient decreasing towards the core of the scaffold. To examine this hypothesis, we repeated the simulations, this time introducing a gradient to the cell death probability that decreases radially towards the core of the scaffold. In turn, the death probability of a cell found in (x_i, y_i, z_i) became,

$$d_{r,i} = p_k \frac{\sqrt{(x_i - x_c)^2 + (y_i - y_c)^2 + (z_i - z_c)^2}}{0.5\sqrt{x_c^2 + y_c^2 + z_c^2}} \tag{5}$$

where $p_k = k\mathrm{d}t$ is the death probability determined by the death rate, k, of the continuum model, and (x_c, y_c, z_c) the center of the space. The radius dependent death probability produced more relevant results with respect to the experiment, increasing the clustering index with respect to treatment (Fig Kc).

Additionally, we discussed about the relationship between mechanisms and morphological patterns. Specifically, in the Discussion section C of the manuscript, we mentioned that the responsible mechanism behind the increasing clustering with respect to time is the advection that biases the movement of the cells towards the increase of signal concentration. On the other side, we would expect that the diffusion mechanism spreads the cancer cells, in turn it would result in less pronounced clustering. To demonstrate the effect of the advection together with the diffusion we performed 2 kinds of simulations, one with low advection constants and high diffusion constants, and one with high advection and low diffusion constants, respectively. In total, 12 simulations for each of the 2 cases were performed. Then, we used the resulted cell distributions to perform the CSR test. Finally, we calculated the are under the curve of the K-function (AUC_K), and we compared it to the values of the diffusion and advection constants. The results presented in Fig N show that the combination of low advection and high diffusion constants produced considerably lower AUC_K values than those with high advection and low diffusion constants. The statistical significance of the resulted AUC_K between these two cases was calculated using the Kruskal-Wallis paired test, and the p-value was found to be 5.4×10^{-5}.

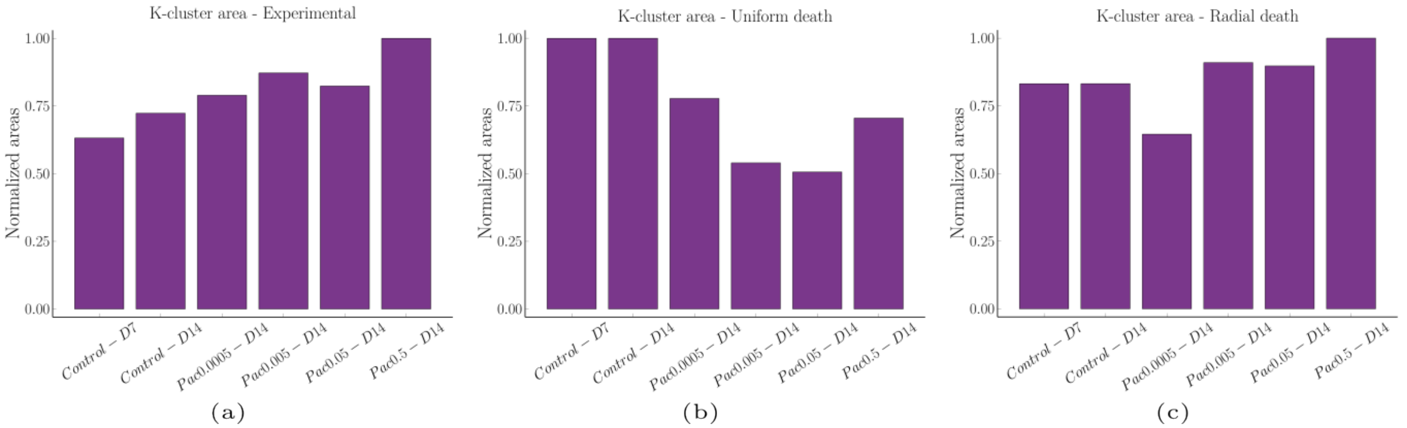

Fig K Model validation analysis. Clustering abundance for (a) Experiments, (b) Simulations with uniform death probability across the space, and (c) Simulations with a radial decrease in cell death probability towards the center of the space.

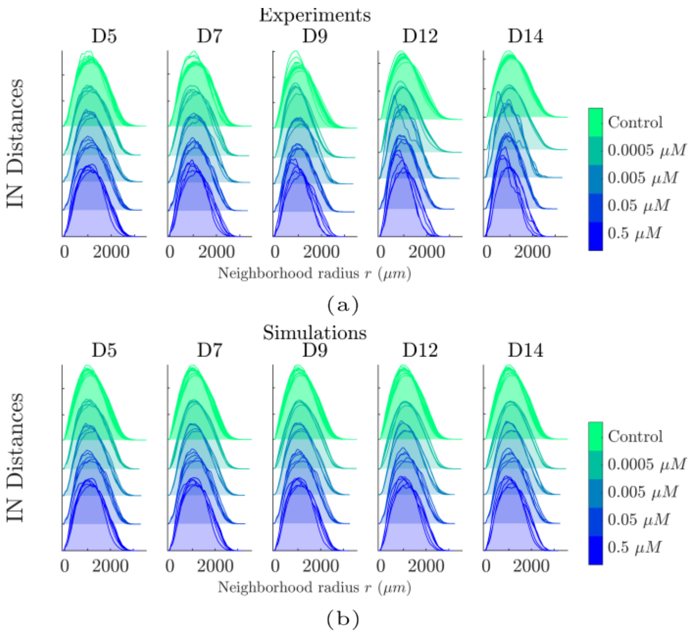

Fig L Spatial analysis. Inter-Nucleic Euclidean distance distributions between (a) experiments, and (b) simulations across time. Overall, the distributions remain stable across time and treatment condition in both experiments and simulations.

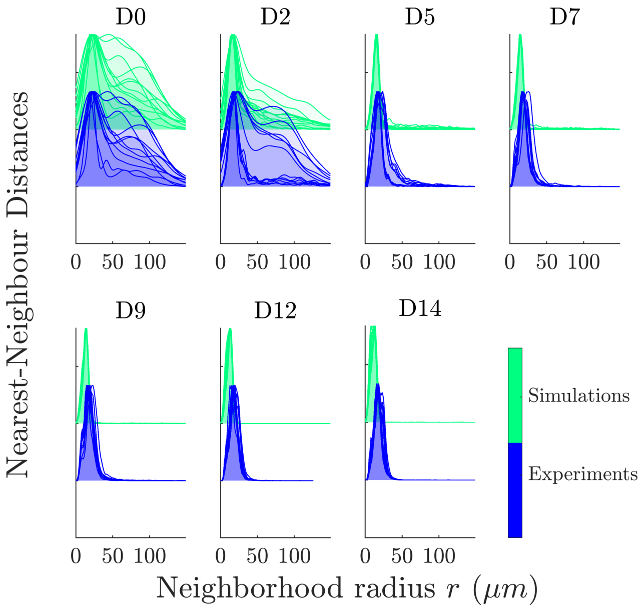

Fig M Spatial analysis. Nearest-Neighbour Euclidean distance distributions of the non-treatment conditions across time. The NN distances initially formed wide distributions that gradually tended to become narrower around lower neighbourhood radii values with respect to time, across all samples, with similar characteristic peaks at ∼15 µm.

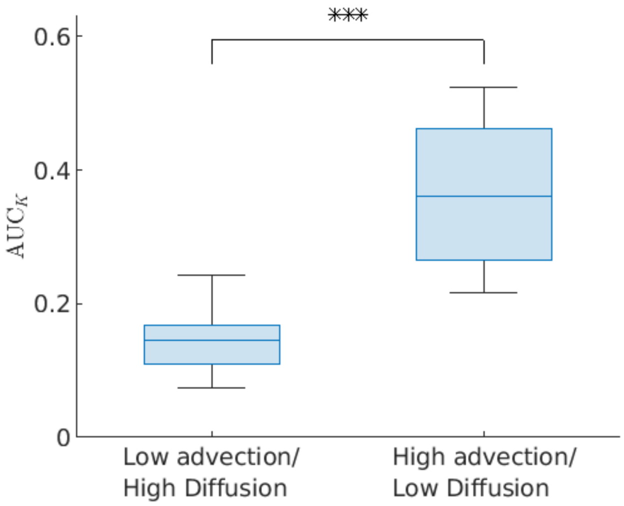

Fig N Spatial analysis. Relationship between diffusion and advection mechanisms with morphological patterns. The K-function was calculated for 12 simulations of two cases; one with low advection (0.0005 ± 0.0004 mm^2/day) and high diffusion (0.0076 ± 0.0003 mm^2/day) constants, and one with high advection (0.0052 ± 0.0002 mm^2/day) and low diffusion (0.00186 ± 0.00009 mm^2/day) constants. The area under the resulted K-function (AUC_K) was then calculated, and it was found that the combination low advection and high diffusion constants produced a considerably lower AUC_K than the combination of high advection and low diffusion constants. The statistical significance of this result was calculated using the Kruskal-Wallis test, and it was found that p-value$\approx 5.4 \times 10^{-5}$.

References

1. Friedl P, Mayor R. Tuning collective cell migration by cell–cell junction regulation. Cold Spring Harbor perspectives in biology. 2017;9(4):a029199.

2. Crowley LC, Scott AP, Marfell BJ, Boughaba JA, Chojnowski G, Waterhouse NJ. Measuring cell death by propidium iodide uptake and flow cytometry. Cold Spring Harbor Protocols. 2016;2016(7):pdb—-prot087163.

3. Jianye C, Yi-Chu C. Transitional Markov Chain Monte Carlo Method for Bayesian Model Updating, Model Class Selection, and Model Averaging. Journal of Engineering Mechanics. 2007;133(7):816–832. doi:10.1061/(ASCE)0733-9399(2007)133:7(816).

4. Hadjidoukas PE, Angelikopoulos P, Papadimitriou C, Koumoutsakos P. Π4U: A high performance computing framework for Bayesian uncertainty quantification of complex models. Journal of Computational Physics. 2015;284:1–21. doi:https://doi.org/10.1016/j.jcp.2014.12.006.
