# Supplementary material for: Cancer cell sedimentation in 3D cultures reveals active migration regulated by self-generated gradients and adhesion sites
Source: PLoS Comput Biol. 2024 Jun 11;20(6):e1012112. doi: 10.1371/journal.pcbi.1012112 (PMC11195982; doi:10.1371/journal.pcbi.1012112)
Supplement: Attachment — Submitted filename: PLoS_CompBio_Peer_Review_2023.pdf [file pcbi.1012112.s002.pdf]

This manuscript describes an integrated experimental/theoretical study investigating the mechanisms behind 3D cell sedimentation within hydrogels. The authors expose breast cancer cells to Paclitaxel, an inhibitor of microtubule assembly, to argue that the largely vertical migration of cells is due to cell-generated forces rather than gravity or ECM compression. Bulk RNA sequencing of cells in control and treatment settings further suggest that this anisotropic movement relies on pathways related to collective behavior. From these observations, the authors hypothesize that such movement arises from chemotactic leader-follower processes such as those seen during wound healing. In this scenario, cells at the bottom of the gel would adhere to the underlying glass substrate and secrete a chemical signal attracting cells near the top of the assay. The authors examine this idea within a theoretical setting by fitting and simulating a hybrid mathematical model based on those for wound healing. The authors find that their *in silico* framework yields results similar to those observed *in vitro*. The authors conclude by performing a series of *in vitro* experiments that aim to corroborate their chemotactic hypothesis. These include tests in which cells are seeded in a less adhesive agarose gel, in addition to those in which cancer cells are co-cultured with fibroblasts. In these latter two experiments, cells do not travel as far as they do in Matrigel.

This paper is not yet acceptable for publication. The authors do not provide adequate experimental evidence to justify their hypothesis that chemotactic migration, rather than some other process, is the main driver of cell behavior. The authors present theoretical evidence that chemotactic movement could be consistent with the data, but only speculate as to how cells specifically communicate with each other. The authors at one point suggest TGF-beta as a possible molecular signal, but there are no experiments presented which prove that knocking down or overexpressing this molecule affects the distance that cells travel. Furthermore, the experiments in this paper do not conclusively rule out other plausible scenarios accounting for cell behavior. For example, the authors argue that ECM compression does not account for the 3D migration of cells but do not provide direct evidence explaining their rationale (in the Supplementary Material they provide graphs of the cell positions and numbers over time, and even acknowledge that compression may occur, but do not present data about the hydrogel itself or justify why they ignore its effect). While the authors' experiments may be consistent with the chemotactic hypothesis proposed here, other interpretations may also be supported. For instance, it may be that cells actively move away each other due to contact inhibition of locomotion, which can produce anisotropic collective movement in other biological contexts (see Szabó 2016, *J. Cell Biol.*; 2019, *PLoS Comput. Biol.*). Given that these authors claim in their manuscript that the active migration is mainly regulated by adhesion and self-generated gradients, they should present more *in vitro* evidence confirming this.

There also are several, more minor, areas in which this manuscript can improve. There are a handful of recent references related to the mathematical modeling of cancer and tumor spheroids which the authors may find useful to include in their next draft (see the list of minor comments below). Additionally, there are several passages and sentences with unclear meaning, which the authors should revise (see the list of minor comments below). Overall, however, the manuscript is well written.

Below please find a list of major and minor comments that should be addressed.

List of Major Comments:

1. Line 154: What experimental evidence is there for a chemotactic molecule? What is the biological justification for this assumption? The authors should provide experimental evidence supporting the assumptions underlying their mathematical model.
2. Lines 272-275: The authors state that "in presence of compression we would observe sedimentation" but also that it "is an inadequate condition for sedimentation". This is a bit confusing / unclear, as these statements appear to be contradictory for this reviewer. Can the authors revise these lines and clarify their meaning?
3. Lines 272-275: The supplementary figures the authors cite in these lines provide information on the distance of cells from the bottom of the assay, but not on the hydrogel itself. Presumably this is why the authors cannot rule out the possibility that ECM compression may occur, but what is their argument / reasoning for ignoring it for the rest of their manuscript?
4. Supplementary Table S.1-S.5, Supplementary Figure S.8: It seems that, for a lot of parameter distributions, the average values are very close to the boundaries of the prior distribution indicated in the tables. Furthermore, the range of parameter values for some variables appears quite small. Both observations suggest that MCMC chains may not actually have converged. Have the authors checked that expanding the boundaries of their priors leads to similar parameter estimates? That is, if you increase the size of the parameter space that can be explored by MCMC, does your posterior distribution become significantly different?
5. Line 295: Why have the authors decided that it is specifically leader-follower migration that is the cause of collective behavior in this context? Other modes of collective migration are possible: e.g., sheet migration, contact inhibition of locomotion, or border cell / cluster migration. It may not be the case that these cancer cells are undergoing leader-follower migration. The authors should comment on whether these other forms of migration could play a role, and whether their data are also consistent with these alternative modes of migration. References include

- Rørth P (2009) Collective cell migration. Annu Rev Cell Dev Biol 25:407–429, DOI 10. 1146/annurev.cellbio.042308.113231

- Shellard A, Mayor R (2020) Rules of collective migration: From the wildebeest to the neural crest. Phil Trans R Soc B 375:20190387, DOI 10.1098/rstb.2019.0387

- Shellard A, Szabó A, Trepat X, et al. (2018) Supracellular contraction at the rear of neural crest cell groups drives collective chemotaxis. Science 362(6412):339–343, DOI 10.1126/ science.aau3301

- Horne-Badovinac S, Bilder D (2005) Mass transit: Epithelial morphogenesis in the Drosophila egg chamber. Dev Dyn 232(3):559–574, DOI 10.1002/dvdy.20286

6. Figure 3: In panel (a), it appears that the MCMC distributions are very close to the boundaries of the prior distributions listed in the supplementary material. This suggests that the MCMC chain may not have converged, or that the distribution itself is much

more complicated than the authors have anticipated. Have the authors collected metrics that verify convergence of the chains? Alternatively, have they widened the boundaries of their prior distributions and verified that they obtain similar parameter estimates?

7. Section G (Lines 376 – 384): Could an alternative interpretation of this experiment be that cells die on the agarose, and it is the loss of this population pressure that prevents them from migrating downwards into the gel? It is unclear to this reviewer whether such alternative explanations are ruled out by this experiment.
8. Lines 394-395: Can the authors exclude the possibility that fibroblasts physically prevent or "block" the cancer cells from moving downwards? Do the authors have direct evidence that it is molecular secretions by fibroblasts that cause the cells to migrate less?
9. Lines 410-411: Did the authors examine the stiffness of their gels, and whether there actually is a durotactic gradient?

List of Minor Comments:

1. Line 35: Other, more recent, references relating to the mathematical modeling of cancer include

- Byrne, H. Dissecting cancer through mathematics: from the cell to the animal model. *Nat Rev Cancer* **10**, 221–230 (2010). https://doi.org/10.1038/nrc2808
- Sfakianakis, Nikolaos, and Mark AJ Chaplain. "Mathematical modelling of cancer invasion: A review." *International Conference by Center for Mathematical Modeling and Data Science, Osaka University*. Singapore: Springer Singapore, 2020.
- Bull, Joshua Adam, and Helen Mary Byrne. "The hallmarks of mathematical oncology." *Proceedings of the IEEE* 110.5 (2022): 523-540.

The authors may find these useful to include in their next draft (but are not obligated to include them by any means).

2. Line 38: A more recent review of discrete modeling of tumor spheroids can be found in

- J. Metzcar, Y. Wang, R. Heiland, and P. Macklin. A review of cell-based computational modeling in cancer biology. JCO Clin. Cancer Inform., 2:1–13, 2019

References for continuum models include

- Walker, Benjamin J., et al. "Minimal Morphoelastic Models of Solid Tumour Spheroids: A Tutorial." *Bulletin of mathematical biology* 85.5 (2023): 1-35.

- -Bull JA, Mech F, Quaiser T, Waters SL, Byrne HM (2020) Mathematical modelling reveals cellular dynamics within tumour spheroids. PLOS Computational Biology 16(8): e1007961. https://doi.org/10.1371/journal.pcbi.1007961

3. Lines 47-56: Other references that could also be included in this paragraph are:

- Lima EABF, Faghihi D, Philley R, Yang J, Virostko J, et al. (2021) Bayesian calibration of a stochastic, multiscale agent-based model for predicting *in vitro* tumor

growth. PLOS Computational Biology 17(11): e1008845. https://doi.org/10.1371/journal.pcbi.1008845

- Turnover Modulates the Need for a Cost of Resistance in Adaptive Therapy. Maximilian A.R. Strobl; Jeffrey West; Yannick Viossat; Mehdi Damaghi; Mark Robertson-Tessi; Joel S. Brown; Robert A. Gatenby; Philip K. Maini; Alexander R.A. Anderson. *Cancer Res* (2021) 81 (4): 1135–1147. https://doi.org/10.1158/0008-5472.CAN-20-0806

4. Reference 39: The DOI for this reference is missing, and should be https://doi.org/10.1016/j.jtbi.2017.10.032
5. Lines 106-107: Does the macroscopic behavior of the cells change when more of them are added (that is, if you have 1000 cells do they behave the same as assays with 10,000 or 100,000 cells? Was a control performed to check on this possibility?
6. Equation (1): According to this model, cancer cells are advected depending on how much space is available -- i.e., there is no advective flux without cells ($u = 0$) or when they take up the whole space ($u = 1$). However, why isn't a similar assumption made for the diffusive flux (the first term on the right-hand side of the equation)? Wouldn't a similar density-dependent approximation be more consistent with the authors' other assumptions?
7. Equation (1): Is treatment always being applied in these experiments? It would be helpful to state whether k is always nonzero.
8. Equation (2): The authors state this is a cell-induced gradient model, but the chemotactic molecule, f, can only diffuse through the domain (i.e., there is no term in the equation that describes its uptake / removal by the cancer cells). Can the authors comment on this?
9. Reference 61: The DOI appears to link to the wrong webpage.
10. Equation (7): Have the authors ensured that their time steps always produce non-negative probabilities? What are the parameters used?
11. Equation (9): Do the authors also estimate the value of the variance for each dataset (the sigma_d parameters)? Or do they fix these parameters a priori? If so, what was their method for doing so?
12. Reference 67: The DOI links to the wrong webpage.
13. Supplementary Figure S.8: It appears several parameters are correlated – can the authors comment on what this might mean, e.g., if this suggests there are non-dimensional quantities that may be important in studying sedimentation phenomena?
14. Line 295: It seems the authors mean to cite Supplementary Fig S.4, not Supplementary Fig S.5?
15. Section E (Lines 300-319): The authors hypothesize a leader-follower model, but their mathematical equations instead assume a homogeneous cell population. Is the discussion of leader-follower behavior necessary for their presentation?
16. Figure 3: Panel (b): The graphs are difficult to distinguish – it may be more useful to look at the time evolution of a 2D cross section of these data, rather than the full 3D plot.
17. Figure 3: Panel (b): Which colors / graphs correspond to the in vitro results and which to the in silico ones? The caption is unclear.
18. Lines 354-356: The authors should comment on mismatches between the simulated and experimental data, especially in panel (b) where it appears that the mathematical model

performs worse at estimating the population size at later times. Would a better quantification of this error correspond to the sum of squared residuals?

19. Line 361-362: What are the statistical tests the authors performed to make sure that the distributions are similar? KS test, Mann-Whitney U-test, etc?
20. Line 370-371: The meaning of the end of this sentence is difficult to decipher. Can the authors revise it to improve clarity?
21. Line 374-375: Again, it is unclear what the authors mean in this final sentence. The authors should revise this to improve clarity.
22. Figure 4: In panel (a), what do the blue / red colors correspond to? How many realizations of the hybrid model were generated to produce the results in these graphs? If just a single realization was used, can the authors justify why, especially since their model incorporates randomness and thus will not necessarily produce the same results each iteration? If the authors did use multiple realizations, how did they decide on the number of realizations to simulate?

23. Figure 4: Panel (c): The authors should consider using a smoother colorbar than the blue-red one, as it can sometimes lead to misleading results (https://www.kennethmoreland.com/color-advice/)

24. Figure 4: Panel (e): Can the authors comment on why the K-function appears to decrease at larger radii? Is this expected?

25. Figure 4: In all graphs, what do the shaded areas correspond to? Is this the range of results, the mean plus/minus a 95% CI, or something else?

26. Supplementary Fig. S.12: This graph is confusing: it may be simpler to depict this as a bar chart with y-values equal to the AUC_K, and x-values corresponding to "high diffusion, low advection" and "low diffusion, high advection", for instance.
