# Supplementary material for: Cancer cell sedimentation in 3D cultures reveals active migration regulated by self-generated gradients and adhesion sites
Source: PLoS Comput Biol. 2024 Jun 11;20(6):e1012112. doi: 10.1371/journal.pcbi.1012112 (PMC11195982; doi:10.1371/journal.pcbi.1012112)
Supplement: Attachment — Submitted filename: response_letter_plos_cb__Copy.pdf [file pcbi.1012112.s003.pdf]

Response letter: PCOMPBIOL-D-23-00758

We would like to thank the editor and reviewers for the time spent on reviewing our manuscript, as well as for their thoughtful comments which have helped us improve the clarity and presentation of our findings. Below are our responses to the comments of the reviewers.

We have also included figures and tables supporting our information. To avoid confusion, when we refer to a table or figure that appears in the manuscript we present it with the prefix M., for the main article, and S. for the supplementary material (e.g. Fig. M.1, Fig. S.7). The figures and tables presented in this response letter do not have any prefix (e.g. Fig. 1, Table 2). Also, we have included the updates made to the manuscript, and we report their location based on the section and line number where they appear. We should note that during the compilation process, the submission website produces 2 kinds of line numbers. To avoid any confusion, we refer to the line numbers with the small fonts that appear in both columns.

Reviewer 1

1. Line 154: What experimental evidence is there for a chemotactic molecule? What is the biological justification for this assumption? The authors should provide experimental evidence supporting the assumptions underlying their mathematical model.

1. Reply: We thank the Reviewer for this comment. In this study, we focused on the phenomenon of sedimentation by examining several potential contributors including passive mechanisms such as gravity, ECM compression, and active mechanisms such as chemotaxis. The results of the paclitaxel treatment experiments suggest that gravity and ECM compression are not sufficient for sedimentation, because sedimentation would have occurred in the presence of treatment if this was the case. Additionally, the RNA-seq experiment which examined the differential expression of genes in presence and absence of treatment revealed upregulated mechanisms of active migration related to chemotactic movement during the control conditions. Hence, based on these results and the fact that MDA-MB-231 cells are naturally adherent, we formulated and examined the hypothesis of self-generated chemotactic gradients emerging from cells attached to sites of increased adhesion, e.g. the glass bottom. The simulation results further supported this hypothesis by showing that the behaviour observed in the experiments is reproduced under the examined model and within acceptable levels of error (average NRMSE $< 40\%$).

Unfortunately, we were not able to identify the molecule responsible for the chemotactic migration since this process is experimentally difficult and lengthy, and it was not possible to perform within the timeframe provided for the reviews. We apologize for this.

Instead, we tried to further validate our hypothesis by performing two additional experiments that further challenge the roles of chemotactic signalling (TNBC-CAFs co-cultures) and adhesion (3D cultures on agarose coated bottom).

Overall, our results do not conclusively support that chemotaxis was the sole contributor for this migration. However, they provided robust evidence for its presence. Therefore, we have made the following changes to the manuscript text, aiming to clarify this point:

Abstract; line 17 *whereby migration was likely to be regulated by the MAPK and TGF-β pathways*

Results; lines 142-144 *Leader cells orient and move based on stimuli such as soluble factors. An example of soluble factor diffusing in the extracellular space is TGF-β although it was not examined in this study.*

Results; line 147 *Acknowledging that leader cells move based on signals*

Results; lines 151-152 *Second, the cells attached to the glass secrete signals*

2. Lines 272-275: The authors state that "in presence of compression we would observe sedimentation" but also that it "is an inadequate condition for sedimentation". This is a bit confusing / unclear, as these statements appear to be contradictory for this reviewer. Can the authors revise these lines and clarify their meaning?

2. Reply: We thank the reviewer for this comment. We apologize for the confusion. We have made the necessary adjustments to clarify that compression can be responsible for sedimentation, however it was not observed in our experiments.

Results; lines 111-114 *In the presence of compression we would have observed sedimentation in both treated and untreated samples... ... Supplementary Fig. S.1 and S.2 suggest that ECM compression may have occurred to some extent; however, its magnitude was insufficient for sedimentation.*

3. Lines 272-275: The supplementary figures the authors cite in these lines provide information on the distance of cells from the bottom of the assay, but not on the hydrogel itself. Presumably this is why the authors cannot rule out the possibility that ECM compression may occur, but what is their argument / reasoning for ignoring it for the rest of their manuscript?

3 Reply: Here we should note that the hydrogel was attached to the bottom of the space. The cells were uniformly distributed in the hydrogel at the beginning of the experiment. Indeed, we could not measure the distance of the hydrogel from the bottom, because the hydrogel is transparent, and it was not visible because the 3D culture was immersed in a liquid medium. Although it is reasonable to expect that the hydrogel exhibited some compression over time, we concluded that this compression was not sufficient to drive the cells towards the bottom, because this would have occurred in both the control as well as the treatment samples. Since sedimentation did not occur in the treatment samples, compression and gravity were not the main reasons for the observed sedimentation.

4. Supplementary Table S.1-S.5, Supplementary Figure S.8: It seems that, for a lot of parameter distributions, the average values are very close to the boundaries of the prior distribution indicated in the tables. Furthermore, the range of parameter values for some variables appears quite small. Both observations suggest that MCMC chains may not actually have converged. Have the authors checked that expanding the boundaries of their priors leads to similar parameter estimates? That is, if you increase the size of the parameter space that can be explored by MCMC, does your posterior distribution become significantly different?

4. Reply: We thank the Reviewer for this comment. Our initial efforts for calibration had broader boundaries for the corresponding priors. During the calibration process, we noticed that some parameters converged to values that were not naturally expected. For

example, the cellular diffusivity converged towards very high values. Upon further investigation, we found that the reason underlying this problem was the attempt of the parameters to fit the noise produced by possibly misaligned samples under the microscope across different time-points, as well as to possible geometric distortions of the Matrigel scaffold. Hence, we reduced the parameter search space to values within an experimentally expected range. For example, the doubling time for the MDA-MB-231 cells is expected to be approximately 3 days, hence we narrowed the parameter search between 0.18 and 0.3 day^{-1}. We restricted the upper bound of the growth rate to 0.3 day^{-1}, because we noticed that the model tended to fit towards higher values when the diffusivity values were high, and this was an indication of fitting to the noise induced by misaligned samples across different time-points. Similarly, the prior parameter boundaries for the treatment samples were narrowed for the same reason. Furthermore, for some parameters such as the signal diffusivity, which we assumed to be unaffected by the treatment, we set prior boundaries that were close to the values found by the parameter estimation in the control conditions, specifically [average - (standard deviation), average + (standard deviation)].

5. Line 295: Why have the authors decided that it is specifically leader-follower migration that is the cause of collective behavior in this context? Other modes of collective migration are possible: e.g., sheet migration, contact inhibition of locomotion, or border cell / cluster migration. It may not be the case that these cancer cells are undergoing leader-follower migration. The authors should comment on whether these other forms of migration could play a role, and whether their data are also consistent with these alternative modes of migration. References include Rørth P (2009) Collective cell migration. Annu Rev Cell Dev Biol 25:407–429, DOI 10. 1146/annurev.cellbio.042308.113231
Shellard A, Mayor R (2020) Rules of collective migration: From the wildebeest to the neural crest. Phil Trans R Soc B 375:20190387, DOI 10.1098/rstb.2019.0387
Shellard A, Szabó A, Trepat X, et al. (2018) Supracellular contraction at the rear of neural crest cell groups drives collective chemotaxis. Science 362(6412):339–343, DOI 10.1126/ science.aau3301
Horne-Badovinac S, Bilder D (2005) Mass transit: Epithelial morphogenesis in the Drosophila egg chamber. Dev Dyn 232(3):559–574, DOI 10.1002/dvdy.20286

5. Reply: We thank the Reviewer for this comment. We agree with the Reviewer that using our experimental setup, it is difficult to distinguish the type of collective migration. Hence our analysis was mostly intended to examine whether the cells migrate collectively or individually.

Sheet migration is mainly found on 2D substrates. It can happen when cells attach to the glass; however, we limited our analysis on the process of sedimentation.

Sprouting and branching are observed mainly as a result of VEGF and EGFL7 stimulation. They has been observed in Matrigel cultures. Although our analysis was not exhaustive, we did not identify a clear pattern of downregulation of VEGF in our differential gene expression analysis. EGFL7 exhibited some level of reduction, however its behavior was highly variable. Thus, we cannot conclusively state whether sprouting and branching were present in our experiments (Fig. 1a).

Multicellular streaming was likely not present, as we did not find any evidence of EMT [1]. On the other hand, contact inhibition of locomotion could have been possible, as Rhoa

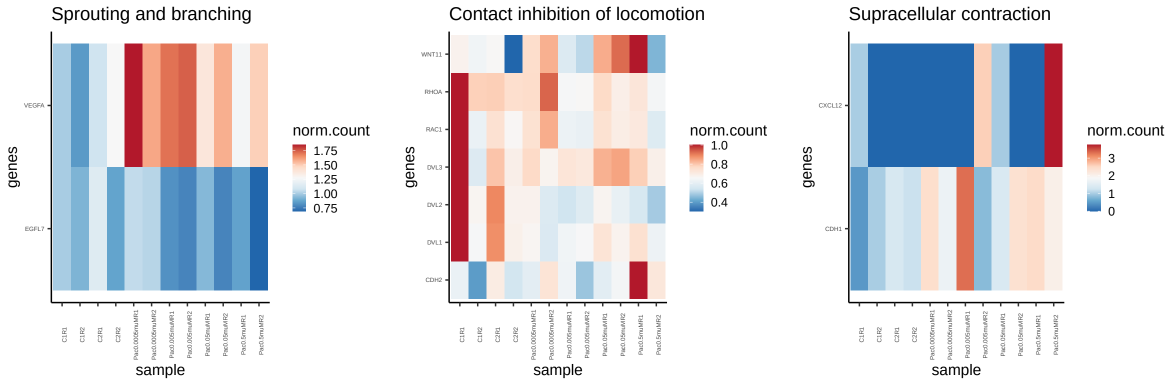

(a) (b) (c)

Figure 1: Alternative possible mechanisms of migration. Genes involved in a) sprouting and branching, b) contact inhibition of locomotion, c) supracellular contraction. C1 and C2 correspond to the control samples. The treatment samples are denoted with Pac following the dose of the treatment in μM.

and Rac1 exhibited high levels of expression in the control samples – an indicator of this type of migration (Fig. 1b) [2, 3]. Finally, there was no evidence that supracellar contraction was present in our study as CXCL12 (SDF1) was not expressed in the control samples (Fig. 1c) [4]. Overall, to address this comment, we have made the following changes to the manuscript.

Discussion, section Alternative mechanisms of migration; lines 283-292 *The differential gene expression analysis provided evidence for collective migration that may have been consistent with the leader-follower mechanism. Nevertheless, our analysis cannot provide conclusive evidence for this mechanism, as this would have required dense time-lapse microscopy data focusing on the required proteins. Thus, we examined the presence of other types of migration. Some candidates are sprouting and branching [1], multicellular streaming [1], contact inhibition of locomotion [2, 3], and supracellular contraction [4] (Supplementary Fig. S.6). Among these mechanisms, we found that contact inhibition of locomotion can contribute to cell migration as Rhoa and Rac1, which are responsible for cell polarity and participate in this type of migration [2, 3], exhibited high levels of expression in the control samples.*

6. Figure 3: In panel (a), it appears that the MCMC distributions are very close to the boundaries of the prior distributions listed in the supplementary material. This suggests that the MCMC chain may not have converged, or that the distribution itself is much more complicated than the authors have anticipated. Have the authors collected metrics that verify convergence of the chains? Alternatively, have they widened the boundaries of their prior distributions and verified that they obtain similar parameter estimates?

6. Reply: We thank the Reviewer this comment. The employed TMCMC algorithm stops automatically when the chains have converged, and in our case it converged after a number of generations. Similar to comment # 4, initially, we tried using broader prior calibration boundaries; however, the model tended to fit noise generated by sample misalignments or by geometric distortions producing unreasonably large diffusivity values. Hence,

we narrowed the calibration boundaries within a more physiologically plausible range.

7. Section G (Lines 376 – 384): Could an alternative interpretation of this experiment be that cells die on the agarose, and it is the loss of this population pressure that prevents them from migrating downwards into the gel? It is unclear to this reviewer whether such alternative explanations are ruled out by this experiment.

7. Reply: We thank the Reviewer for this comment. We should clarify that the cells were not in contact with the agarose, and they were encapsulated with Matrigel. They were also immersed into a liquid medium; hence, they had access to nutrients. The role of the agarose was only to "hide" the glass bottom from the cells. Hence, we believe that the absence of adhesion was responsible for cell death.

8. Lines 394-395: Can the authors exclude the possibility that fibroblasts physically prevent or "block" the cancer cells from moving downwards? Do the authors have direct evidence that it is molecular secretions by fibroblasts that cause the cells to migrate less?

8 Reply: We agree with the Reviewer that the mixing between cells and fibroblasts occurring during the later stages of the experiment may have physically blocked cell migration and sedimentation. However, during the initial stages of the experiment, we observed both fibroblast network assembly as well as and their invasion in the ECM, which we believe was due to signalling with the cancer cells. For this comments, we made the following changes in the manuscript.

Discussion, Section E, lines; 326-331 *In contrast with the previous experiment, the presence of fibroblasts likely induced migration signals and increased adhesion leading to cell-fibroblast mixing across the outer surface of the Matrigel scaffolds. In turn, the cancer cells exhibited less pronounced migration towards the bottom and maintained elevated positions in the 3D space (Fig. 6). At this point we should not exclude the possibility that fibroblasts physically blocked cell migration, however their aggregation with the cancer cells can be due to signalling.*

9. Lines 410-411: Did the authors examine the stiffness of their gels, and whether there actually is a durotactic gradient?

9. Reply: We thank the Reviewer for this comment. Unfortunately, due to the small scale of the experiment, we were not able to measure the stiffness of the ECM. However, we would like to point out that durotaxis may also occur in single cells [2]. Therefore, we have changed the corresponding reference in the original manuscript [2] to one that concerns both collective and individual durotactic migration [3]. With regards to the interface between the glass surface and the Matrigel ECM, even if this interface exhibits a lower resistance (which may not necessarily be the case - as we would normally expect uniform properties of the ECM across its volume), it is also questionable whether cells that are e.g. 1 mm far from it can sense this reduced resistance. On the other hand, previous studies [4] have shown that cells can move using self generated chemotactic gradients over long distances. However, as we agree that the adhesion properties of the glass bottom are an important aspect, we further investigated the effect of these properties on cell migration using the Matrigel on agarose experiment.

List of minor comments:

1. Line 35: Other, more recent, references relating to the mathematical modeling of

cancer include Byrne, H. Dissecting cancer through mathematics: from the cell to the animal model. Nat Rev Cancer 10, 221–230 (2010). https://doi.org/10.1038/nrc2808 Sfakianakis, Nikolaos, and Mark AJ Chaplain. "Mathematical modelling of cancer invasion: A review." International Conference by Center for Mathematical Modeling and Data Science, Osaka University. Singapore: Springer Singapore, 2020. Bull, Joshua Adam, and Helen Mary Byrne. "The hallmarks of mathematical oncology." Proceedings of the IEEE 110.5 (2022): 523-540. The authors may find these useful to include in their next draft (but are not obligated to include them by any means).

1. Reply: We have included the suggested references. Thank you.

2. Line 38: A more recent review of discrete modeling of tumor spheroids can be found in J. Metzcar, Y. Wang, R. Heiland, and P. Macklin. A review of cell-based computational modeling in cancer biology. JCO Clin. Cancer Inform., 2:1–13, 2019

References for continuum models include

Walker, Benjamin J., et al. "Minimal Morphoelastic Models of Solid Tumour Spheroids: A Tutorial." Bulletin of mathematical biology 85.5 (2023): 1-35.

Bull JA, Mech F, Quaiser T, Waters SL, Byrne HM (2020) Mathematical modelling reveals cellular dynamics within tumour spheroids. PLOS Computational Biology 16(8): e1007961. https://doi.org/10.1371/journal.pcbi.1007961

2. Reply: We have included the suggested references. Thank you.

3. Lines 47-56: Other references that could also be included in this paragraph are: Lima EABF, Faghihi D, Philley R, Yang J, Virostko J, et al. (2021) Bayesian calibration of a stochastic, multiscale agent-based model for predicting in vitro tumorgrowth. PLOS Computational Biology 17(11): e1008845. https://doi.org/10.1371/journal.pcbi.1008845

Turnover Modulates the Need for a Cost of Resistance in Adaptive Therapy. Maximilian A.R. Strobl; Jeffrey West; Yannick Viossat; Mehdi Damaghi; Mark Robertson-Tessi; Joel S. Brown; Robert A. Gatenby; Philip K. Maini; Alexander R.A. Anderson. Cancer Res (2021) 81 (4): 1135–1147. https://doi.org/10.1158/0008-5472.CAN-20-0806

3. Reply: We have included the suggested references. Thank you.

4. Reference 39: The DOI for this reference is missing, and should be https://doi.org/10.1016/j.jtbi.2017.10.032

4. Reply: We have included the DOI. Thank you.

5. Lines 106-107: Does the macroscopic behavior of the cells change when more of them are added (that is, if you have 1000 cells do they behave the same as assays with 10,000 or 100,000 cells? Was a control performed to check on this possibility?

5. Reply: We thank the Reviewer for this comment. For the RNAseq experiment, we had to increase the number of cells, as well as the number of scaffolds in order to achieve the desired amount of RNA extracted from the cells. The behavior of the cells remained similar, exhibiting sedimentation at earlier time-points possibly due to cell crowding.

6. Equation (1): According to this model, cancer cells are advected depending on how much space is available – i.e., there is no advective flux without cells ($u = 0$) or when they take up the whole space ($u = 1$). However, why isn't a similar assumption made for the diffusive flux (the first term on the right-hand side of the equation)? Wouldn't a similar

density-dependent approximation be more consistent with the authors' other assumptions?

6. Reply: The $u(u-1)$ factor in the advection term was mainly added to avoid over-crowding during advection, which can lead to physically unexpected density spikes. Density spiking during advection is a known effect of Keller-Segel type models which can be consistent with bacterial migration; however, it has not been observed in cancer. As a result, we introduced this limiting term to avoid both the spiking behavior as well as numerical instabilities in the solution of the model. We agree that a density dependent diffusion may be more consistent with the model; however, we wanted to keep the model as simple as possible for reasons of simulation performance and numerical accuracy, as the calibration process was time consuming due to the large number of datasets.

7. Equation (1): Is treatment always being applied in these experiments? It would be helpful to state whether k is always nonzero.

7. Reply: The treatment term is zero for the control condition throughout the time-course of the experiment. For the non-treatment conditions, treatment is applied on day 5, hence k is non-zero for $t \geq 5$ days. To save some computational time, in the conditions we restricted our simulations between 5 and 14 days, while in the non-treatment conditions we simulated the entire experiment, i.e. between 0 and 14 days. This did not affect the results, since we had enough time-points to perform calibration considering that the parameters s, D_f were considered unaffected by the treatment and thus their prior boundaries were restricted by their average values $\pm$ standard deviation, as obtained from the calibration for the control experiments.

8. Equation (2): The authors state this is a cell-induced gradient model, but the chemotactic molecule, f, can only diffuse through the domain (i.e., there is no term in the equation that describes its uptake / removal by the cancer cells). Can the authors comment on this?

8. Reply: We thank the Reviewer for this comment. Indeed our initial model included interaction terms between signals and cells; however, we performed a global sensitivity analysis and we found that this term did not contribute significantly to the model output and hence we discarded it. More information can be found in our previous work [5]. To clarify this point, We have made the following changes the manuscript.

Methods; lines 486-488 *In our previous analysis [5], we found that the signal production term in (2) was insensitive to the produced output; hence, we excluded it from the current model.*

9. Reference 61: The DOI appears to link to the wrong webpage.

9. Reply: We have corrected the DOI. Thank you.

10. Equation (7): Have the authors ensured that their time steps always produce non-negative probabilities? What are the parameters used?

10. Reply: We thank the Reviewer for this comment. Indeed, the selection of large time intervals in the temporal grid may result in negative probabilities. Our algorithm takes it into account and adjusts the interval Δt to non-negative values. This is also one of the reasons that make the hybrid model computationally expensive, hence more difficult to calibrate.

11. Equation (9): Do the authors also estimate the value of the variance for each dataset

(the σ_d parameters)? Or do they fix these parameters a priori? If so, what was their method for doing so?

11. Reply: We confirm that the value of the variance σ_d was estimated. Some instances of their posteriors can be found in Fig. S.9.

12. Reference 67: The DOI links to the wrong webpage.

12. Reply: We have corrected the DOI. Thank you.

13. Supplementary Figure S.8: It appears several parameters are correlated – can the authors comment on what this might mean, e.g., if this suggests there are non-dimensional quantities that may be important in studying sedimentation phenomena?

13. Reply: We thank the Reviewer for this comment. It is expected for the parameters D_f and χ to be positively correlated, because they determine the magnitude of sedimentation. For example, when the diffusivity D_f is small, a higher gradient of f is expected compared to a large D_f. As a result, the cells move faster towards the bottom. Furthermore, the balance of the migration speed is determined by χ. When χ is small the cells will move slower, while when it is large, they move faster. On the other hand, when D_f is high, the gradient becomes smaller, the migration speed will be also small, and thus χ will increase to increase the migration speed. Overall, these two parameters have opposite effects. Low D_f increases migration speed, and low χ decreases it. High D_f decreases migration speed, and high χ increases it.

14. Line 295: It seems the authors mean to cite Supplementary Fig S.4, not Supplementary Fig S.5?

14. Reply: We have corrected the citation to Fig. S.4. Thank you for noticing this mistake.

15. Section E (Lines 300-319): The authors hypothesize a leader-follower model, but their mathematical equations instead assume a homogeneous cell population. Is the discussion of leader-follower behavior necessary for their presentation?

15. Reply: We thank the Reviewer for this comment. It is true that the model describes a homogeneous cell population. Based on the structure of the CA, the cells are organized into groups and they can also escape the group and move towards the bottom. Once they reach the bottom or they connect with a cell attached to the bottom, they stop migrating. It is true that the model is more granular than what is postulated by the leader-follower hypothesis. However, the aim of the leader-follower hypothesis is to provide an explanation on how this type of migration was initiated. The cells that were closer to the bottom (i.e. leaders) attached to it and they started secreting signals to indicate a site of favorable attachment. Subsequently, the cells above them (i.e. followers) sensed these signals and they migrated towards them and at the same time they secreted signals creating an avalanche effect and a signalling gradient.

16. Figure 3: Panel (b): The graphs are difficult to distinguish – it may be more useful to look at the time evolution of a 2D cross section of these data, rather than the full 3D plot.

16. Reply: 2D cross-sections may be confusing as they make more visible the sample misalignment. In the supplementary material, we show a few examples of cross-sections from all the examined conditions. The new figure can be found here as Fig. 2.

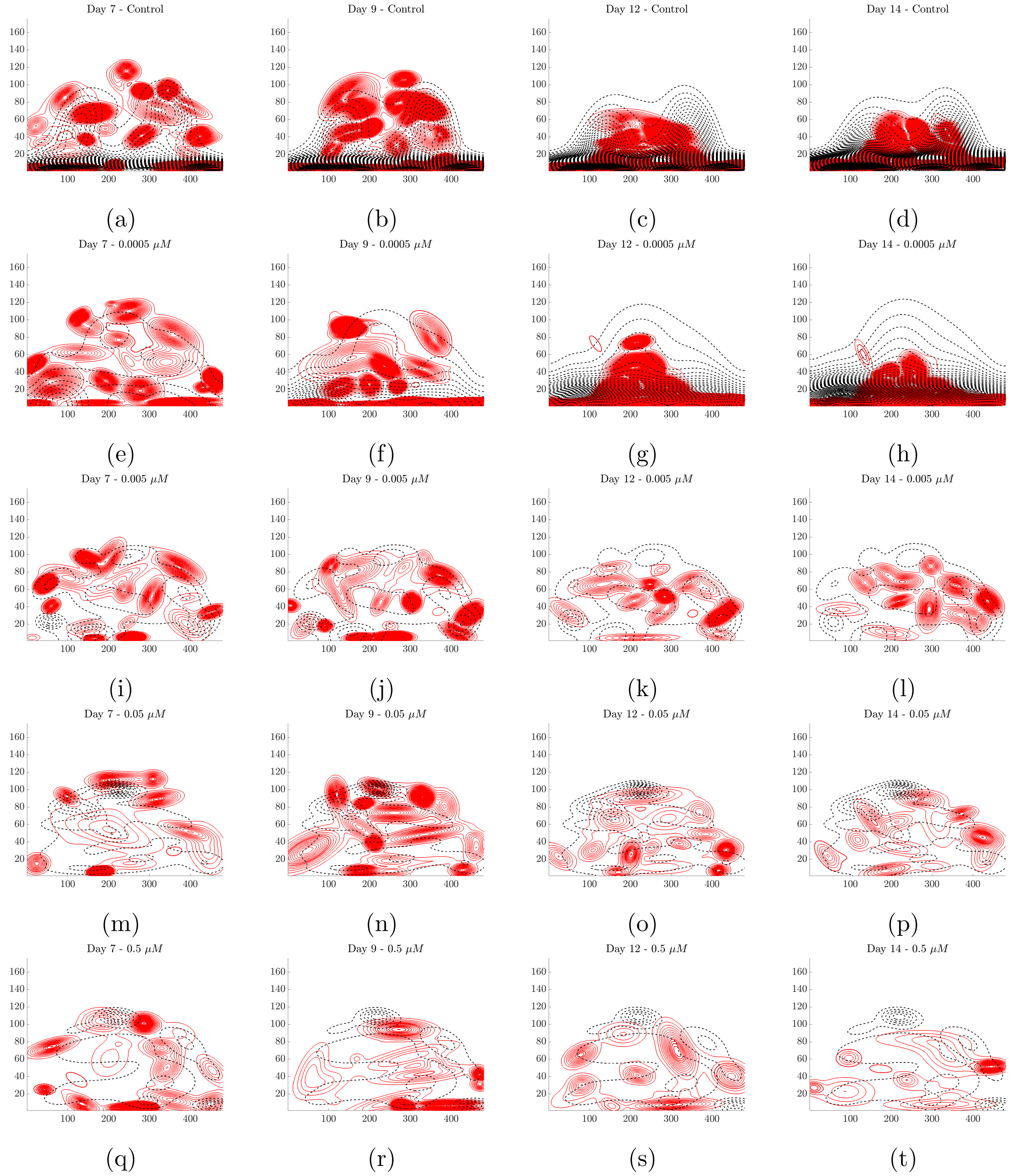

Figure 2: Continuum model calibration. Contour plots for the y-z cross-section at x = 1.25 mm. a)-d) No treatment, e)-h) 0.0005 μM of Paclitaxel, i)-l) 0.005 μM of Paclitaxel, m)-p) 0.05 μM of Paclitaxel, q)-t) 0.5 μM of Paclitaxel for days 7, 9, 12, 14. Red: experiments, Black: simulations.

17. Figure 3: Panel (b): Which colors / graphs correspond to the in vitro results and which to the in silico ones? The caption is unclear.

17. Reply: We have included an explanation for the colors in the caption. Thank you.

Fig. 3b): ... *The green coloured profiles correspond to experiments and the blue to simulations.*

18. Lines 354-356: The authors should comment on mismatches between the simulated and experimental data, especially in panel (b) where it appears that the mathematical model performs worse at estimating the population size at later times. Would a better quantification of this error correspond to the sum of squared residuals?

18. Reply: It is true that the mathematical model performed worse at later timepoints. This was possibly due to adhesion effects that were not taken into account by the mathematical model, although sedimentation was taken into account by the model. This is also reflected on the obtained NRMSE values. We believe that NRMSE is a better (though not perfect) metric for describing the prediction performance of different models, as it takes into account the differences in the sum of squared residuals which may occur for different datasets/models. For instance, in the present study, we can consider the treatment and the no-treatment model as an example. Although the scale of our experiments does not significantly vary across different conditions, the presence of treatment yielded different density profiles compared to the control data; hence, the SSE may not be suitable for comparisons across different conditions.

19. Line 361-362: What are the statistical tests the authors performed to make sure that the distributions are similar? KS test, Mann-Whitney U-test, etc?

19. Reply: We did not perform any statistical test to examine the similarity of the distributions coming from the different calibrations. The distributions exhibited some level of dissimilarity which is expected in the calibration of models with high dimensionality (see [6] and [7] on identifiability). Thus, we combined the posteriors of all the samples from the same condition and as "best" parameter values we selected the average of the posterior for each parameter.

20. Line 370-371: The meaning of the end of this sentence is difficult to decipher. Can the authors revise it to improve clarity?

20. Reply: We have revised the sentence. Below is the correction.

Results; lines 209-211 *The control samples yielded clustered patterns that became more pronounced with respect to time (Supplementary Section S.7, Fig. S.10a), possibly due to cell aggregation at the bottom.*

21. Line 374-375: Again, it is unclear what the authors mean in this final sentence. The authors should revise this to improve clarity.

21. Reply: We have revised the sentence. Below is the correction.

Results; lines 214-216 *Combining these two observations, we conclude that these patterns were possibly due to the radial shrinkage of the spatial cell distributions towards the centroid of the scaffold.*

22. Figure 4: In panel (a), what do the blue / red colors correspond to? How many realizations of the hybrid model were generated to produce the results in these graphs? If just a single realization was used, can the authors justify why, especially since their model incorporates randomness and thus will not necessarily produce the same results each iteration? If the authors did use multiple realizations, how did they decide on the number of realizations to simulate?

22. Reply: Blue corresponds to simulation and red corresponds to experiments. We agree with the Reviewer that stochastic models require multiple simulations to extract an average behaviour. Unfortunately, in the present case we had 32 available datasets, and each simulation of the hybrid model takes approximately 20 minutes. As a result, we needed approximately 11 hours to run 1 iteration per 32 datasets. We tried to restrict our analysis to the control cohort, which contained 12 datasets. The maximum amount of time that we were allowed to use in the computing cluster was 24 hours. Hence, in a single run, we could only perform 6 simulations per dataset for the 12 datasets in a single GPU. Thus, a lot of time and resources were required to run multiple simulations of the hybrid model. However, since the behaviour of the cells in the CA is largely determined by the continuum model, we found that different realizations of the hybrid model with the same parametrization produced similar results. In Fig. 4a-c, we observe that the growth curves, the Inter-Nucleic and Nearest-Neighbour distance distributions remained highly similar across 6 simulations when using the same parametrization and ICs.

23. Figure 4: Panel (c): The authors should consider using a smoother colorbar than the blue-red one, as it can sometimes lead to misleading results (https://www.kennethmoreland.com/color-advice/)

23. Reply: We have changed the colormap to a diverging one. The updated figure is shown in Fig. 6.

24. Figure 4: Panel (e): Can the authors comment on why the K-function appears to decrease at larger radii? Is this expected?

24. Reply: The decrease in the K-function suggest sparsity at larger radii. In our dataset this may be due to the small number of cells compared to the volume within which the K-function was calculated. However, it may be also due to edge effects, although we used edge correction techniques.

25. Figure 4: In all graphs, what do the shaded areas correspond to? Is this the range of results, the mean plus/minus a 95% CI, or something else?

25. Reply: Assuming that the Reviewer refers to Fig. M.4e, the shaded areas correspond to the standard error of the mean (SEM). We have included this information in the caption of Fig. 6e.

26. Supplementary Fig. S.12: This graph is confusing: it may be simpler to depict this as a bar chart with y-values equal to the AUC_K, and x-values corresponding to "high diffusion, low advection" and "low diffusion, high advection", for instance.

26. Reply: We updated the figure using a boxplot, thank you. The new plot can be found in Fig. 3.

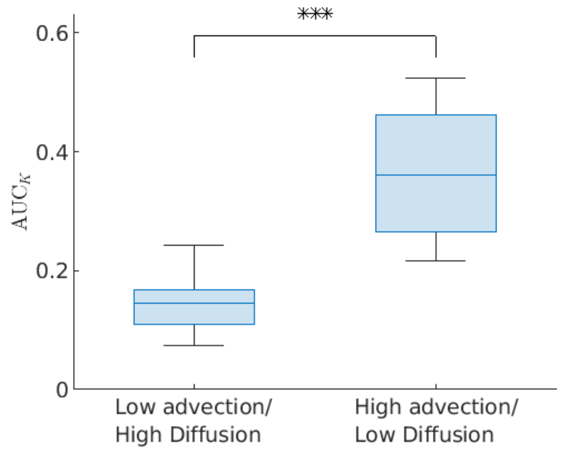

Figure 3: Relationship between diffusion and advection mechanisms with morphological patterns. The K-function was calculated for 12 simulations of two cases; one with low advection and high diffusion constants, and one with high advection and low diffusion constants. The area under the resulting K-function (AUC_K) was then calculated, and it was found that the combination of low advection and high diffusion constants yielded a considerably lower AUC_K than the combination of high advection and low diffusion constants. The statistical significance of this result was calculated using the Kruskal-Wallis test, and it was found that p-value$\approx 5.4 \times 10^{-5}$.

Reviewer 2

This is a well written manuscript combining experiments with mathematical modeling for a better understanding of the sedimentation of cancer cells in 3D cultures. The article contains nice and novel experimental findings, which would make the article suitable even for interdisciplinary journals, beyond specialized computational biology journals. The mathematical models employed are not novel but their combination with the experimental data to provide a better understanding of the mechanisms of cancer cell sedimentation is novel and interesting. I have some specific comments to make.

1. A paragraph in the Methods describing the overall strategy of the study would be helpful. The authors provide briefly a description in the beginning of the Results and throughout the presentation of the results but it is not sufficient. I would suggest to add a separate paragraph in the Methods describing the strategy of the study.

1. Reply: We thank the Reviewer for the overall positive assessment of our work and for this suggestion. In the revised manuscript we provide a brief description in the methods section and we have restructured the manuscript using the following sequence based on Reviewer's # 3 suggestion; Introduction → Results → Discussion → Conclusions → Methods . The following paragraph has been included in the main manuscript.

Methods; lines 382-395 *To examine the phenomenon of sedimentation in 3D cultures, we performed the following experimental and computational modelling steps. First, we performed 3D Matrigel cultures of TNBC cells and we collected spatial data using confocal microscopy across different time-points. To examine the effect of passive migration we performed 3D matrigel cell cultures treated with Paclitaxel, which affects the microtubules of*

the cells, hence inhibiting any possible active migration. Upon rejection of passive migration mechanisms, we performed RNA-seq experiment and differential gene expression analysis to examine the presence of mechanisms responsible of migration. These observations led to the development of a hypothesis of cell sedimentation involving self-generated chemotactic gradient which was examined using a hybrid discrete-continuum mathematical model. The model was calibrated and validated with the experimental data. Finally, to further validate the examined hypothesis we performed two additional experiments that involved cancer cell/fibroblast co-cultures and 3D cultures in agarose coated bottom. These experiment examined the signalling hypothesis and the contribution of sites of high adhesion to the migration. The methods are described in the following paragraphs.

2. The mathematical models are not clearly presented. A schematic of the models along with any boundary and initial conditions is missing. Also a clear presentation of the values of model parameters is missing. There is a reference to what the authors found to be the most critical parameters but a detailed presentation in form of Table(s) showing the parameters, their values, and references from where these values were taken or how they were defined is missing.

2. Reply: The boundary and the initial conditions are shown in Eq. 3, 4, 5. We apologize for neglecting to cite the tables with model parameter in the main manuscript file. The model parameters can be found in Tables S.1-S.5. Samples of the posterior distributions of the model parameters are presented in Fig. S.9. All the parameters were estimated from the experimental data, hence we did not obtain these values from the literature. These tables and the figure are now cited in the main manuscript. Below are the relevant changes made to the manuscript.

Results; lines 175-177 *Approximately 20000 different sets of model parameters were assessed using the Transitional Markov Chain Monte Carlo (TMCMC) method for each of the 32 datasets (Supplementary Section S.4, Tables S.1-S.5, Fig. S.9).*

3. I really like figure 1 but the fonts in panels c and d are too small to read them. The same applies to panels of Figures 3 and 4.

3. Reply: We apologize for this. We have updated Fig. M.1, M.3, M.4. The figures can be found here as Fig. 4, 5, 6.

4. The Discussion is lengthy and repeats the Results. I think it should be shortened significantly and be a discussion of issues that have been discussed previously in the manuscript. Other than these issues, I think it is a good work to be published in PLOS Comp. Biology.

4. Reply: We removed parts of the discussion that seemed to be repetitive, specifically from the Discussion paragraph A. However, we added paragraph C based on the comments made by Reviewer #1. Thank you.

Reviewer 3

The authors study the phenomena of cell sedimentation in which cells tend to undergo downward vertical migration in 3D hydrogel cultures. The authors proposed a model based on active migration based on a chemotactic gradient that is self-generated by the cells.

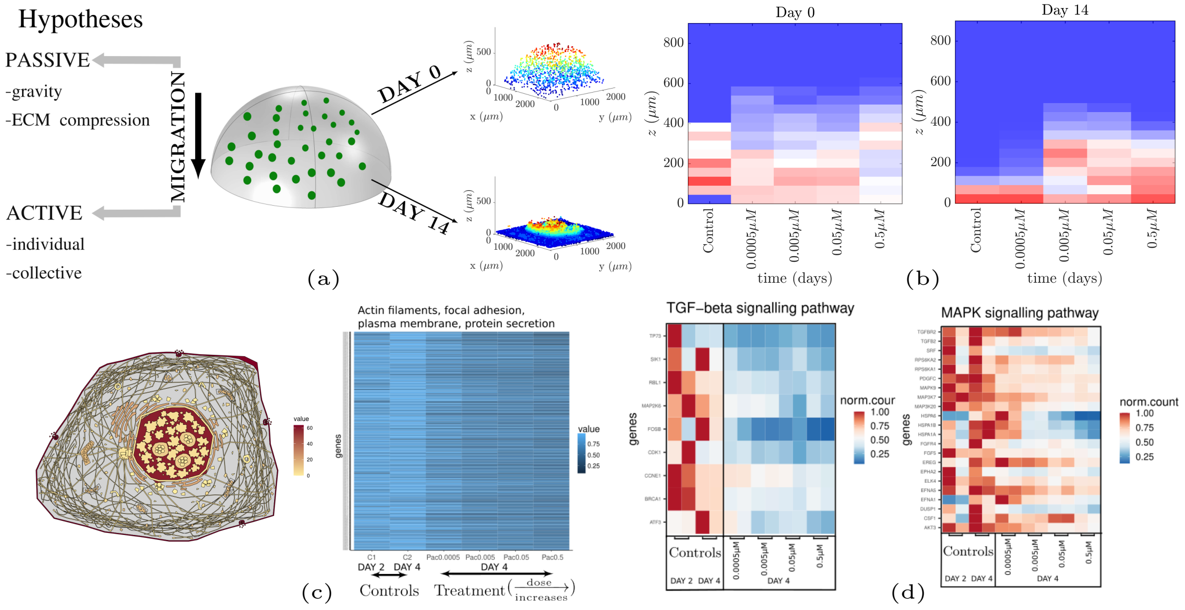

Figure 4: Investigation of migration mechanisms (a) Schematic representation of 3D cultures (center). The cells were uniformly distributed across the semi-ellipsoid on day 0 and actively migrated towards the glass bottom on day 14 (right panel). The examined hypotheses included passive and active migration mechanisms (left). (b) Cell distribution across different cell culture heights on days 0 and 14 in the presence (four different doses) and absence of Paclitaxel. Migration was inhibited for the three highest Paclitaxel doses, ruling out the possibility of passive migration. The glass bottom is located at z=0μm. (c) (left) Cellular anatogram relating the gene expression activity to the corresponding cellular components. (right) Genes related to actin filaments, protein secretion, focal adhesion sites, and plasma membrane exhibited a monotonic decrease in their expression with respect to Paclitaxel dose. These cellular components are also involved in cell migration. (d) TGF-β (left) and MAPK (right) signalling pathways were downregulated as a function of the Paclitaxel dose. The two columns per condition belong to the two corresponding replicates. The inhibition of migration in combination with the downregulation of these pathways suggests that collective cell migration was more likely, as compared to individual migration (i.e., epithelial-to-mesenchymal transition (EMT); Supplementary Fig. S.4).

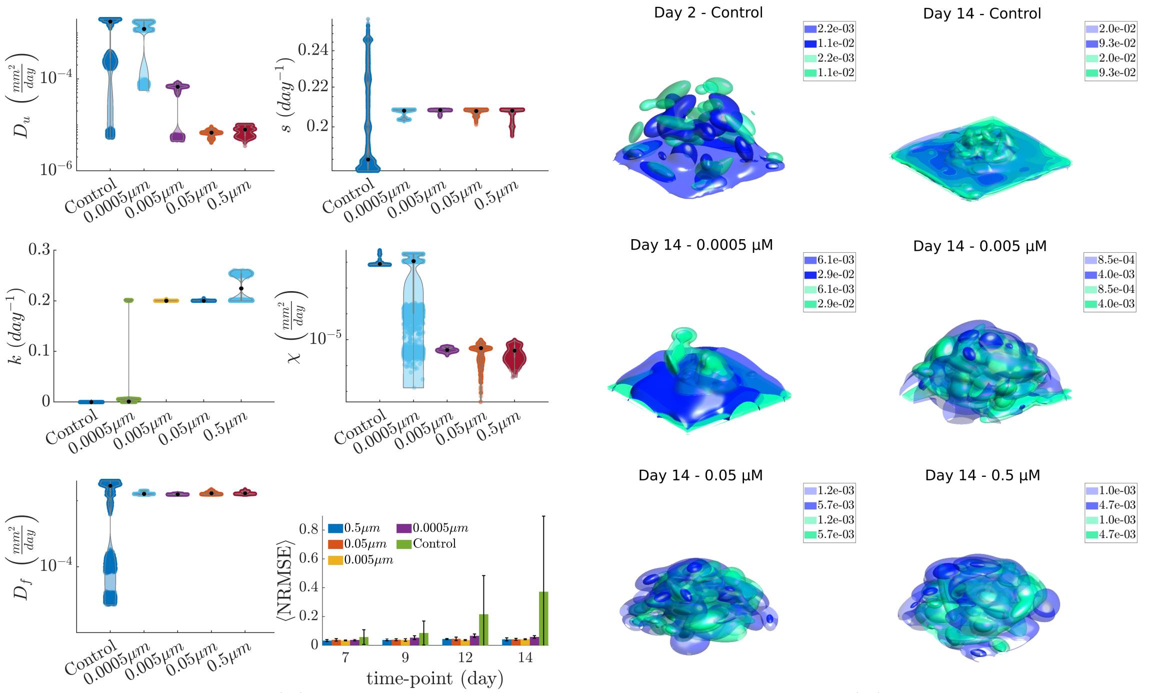

(a) (b)

Figure 5: Continuum model calibration. (a) Posterior marginal distributions of the estimated model parameters for the examined experimental conditions. The NRMSE values for these conditions (bottom right) suggest overall low errors for all conditions, with the model yielding overall higher errors for the non-treatment condition during cell aggregation. (b) Surface plots of the *in vitro* and *in silico* density profiles for representative datasets on days 2 and 14. Paclitaxel was administered on day 5; hence, day 2 was not affected by treatment, while on day 14, we observed a difference in the spatial cell density distributions between the non-treatment and treatment conditions. The distributions remained relatively constant for the three highest doses because cell migration was inhibited, while for the lowest dose and control conditions. the distribution changed due to cell sedimentation. The green coloured profiles correspond to experiments and the blue to simulations.

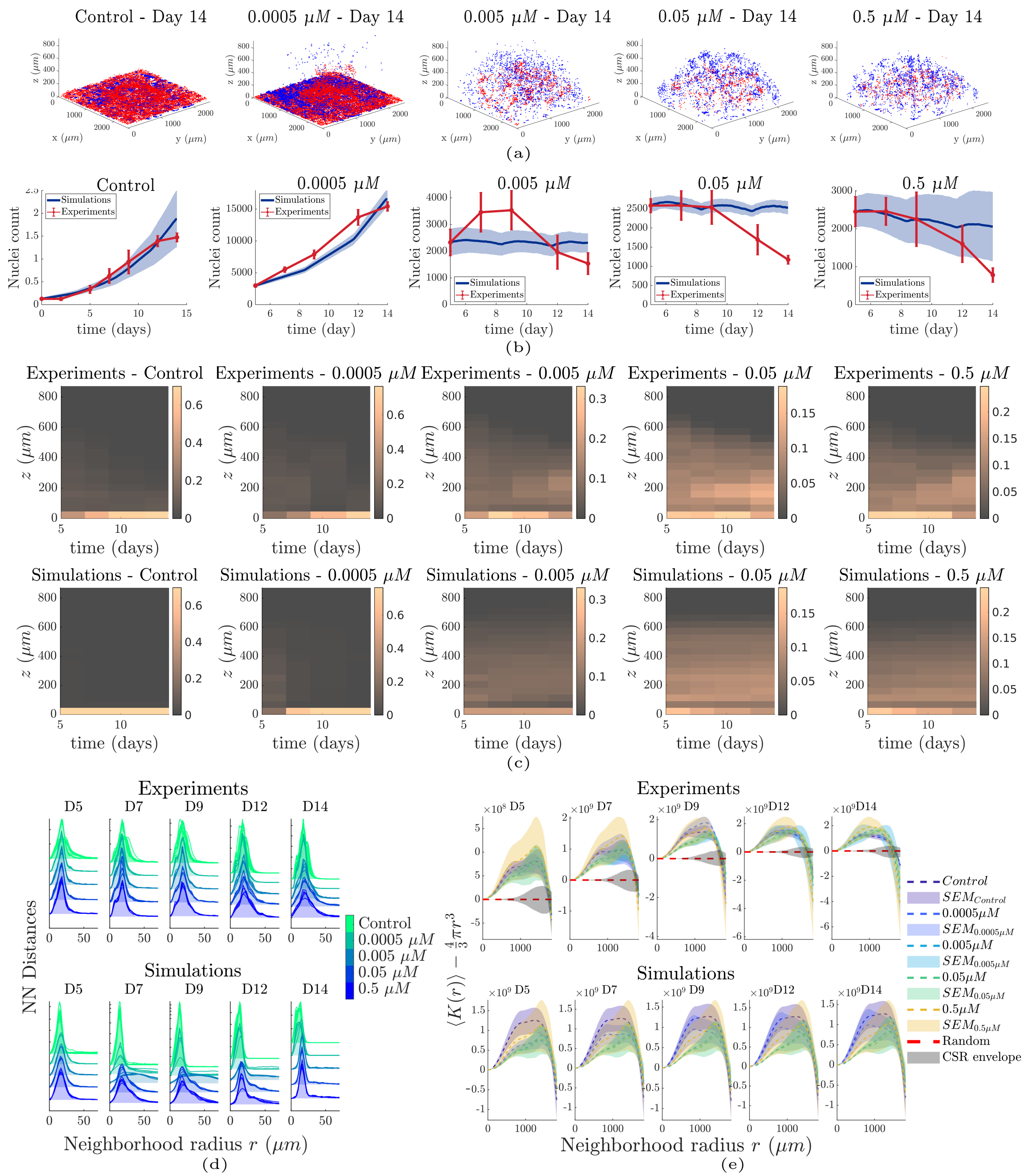

Figure 6: Hybrid modelling results and spatial analysis. (a) 3D plots of cell locations on day 14 for the control and treatment conditions. Red: experiments/Blue: simulations. (b) Predicted and experimentally observed cell numbers. The total cell populationoverall decreased with increasing doses. (c) Histograms of the proportion of cells as a function of position in the culture across time for the experimental (top row) and simulated (bottom row) data, respectively. (d) Nearest Neighbour distances across the examined conditions with respect to time, for the experimental and simulated data. (e) Complete Spatial Randomness test; average values of the K-function across all samples and the corresponding standard error of mean (SEM).

They calibrate a mathematical model using cell culture data and conclude that this provides evidence for their theory.

There are a few aspects of the work that need some clear explanation before I can recommend publication.

Major comments:

1. Why is understanding cell sedimentation important? The only motivation I can find in Lines 50-63 which essential reduce it to "generally undesirable" and typically assume to be gravity based "least resistance". This does not convince me that this is an important problem. I am not saying it isn't, but the authors should state their case more strongly.

1. Reply: We thank the Reviewer for this comment. In the following paragraph, which has been added to the revised manuscript, we provide more information on the importance of sedimentation.

Introduction; lines 71-78 *The understanding of this phenomenon can potentially impact two major research fields. On the one hand, it can lead to better experimental design. In some cases, such as in spheroid formation assays, sedimentation can be an unwanted behavior [8], hence it is important to prevent it if possible. On the other hand, 3D migration assays can lead to important findings on the mechanobiology of cancer progression [9–13]. Another potential utility of sedimentation assays, is that they resemble a 3D analog of the 2D wound healing assays where the cells exhibit their migration potential by converging to the bottom instead of filling the gap in the 2D case.*

2. The other thing I noted is that there does not seem to be any model selection performed and the main evidence against gravity induced migrations is not based on the any underlying model (Section 3A). It concerns me a little that Paclitaxel arrests both active migration and proliferation... could it not be possible that if proliferation was present that sedimentation could be observed? To me it would be desirable to calibrate both the model presented in Section 2H with an alternative representing the gravity induced migration (e.g., no chemotaxis but motility bias base on gravity). The number of parameters will differ in each model so some model selection measure will be required.

2. Reply: We thank the Reviewer for this comment. We agree that it is important to examine different model categories. We apologize that we cannot perform any further calibrations due to lack of computational resources. The calibration process was computationally expensive (4-6 hours per sample, utilizing 16 GPUs) with respect to the examined conditions and we were able to perform only one model calibration. Our future goals include the expansion of the presented computational framework to include model selection techniques, based on previous studies such as the Occam Plausibility algorithm proposed in [6, 14, 15].

3. In the model calibration I do not see the details on the priors, these need to be reported and some exploration of sensitivity to the priors are needed.

3. Reply: We apologize for neglecting to cite the relevant figures, which can be found in the Supplementary material. The model parameters can be found in Tables S.1-S.5. Samples of the posterior distributions of the model parameters are presented in Fig. S.8. All the parameters were estimated from the experimental data, hence we did not obtain these values from the literature. These tables and the figure are now cited in the main manuscript.

The prior boundaries are shown in the headers of the corresponding tables and they follow a uniform distributions (Supplementary Section S.4). Sensitivity analysis was performed in a previous manuscript [5] which focused on the developed computational framework, and showed that all the parameters are sensitive except for a signal interaction which was not included in the model (see also response to Comment # 8 from Reviewer 1). The article [5] is cited in the main manuscript in the Mathematical Model subsection (line 487) of Methods. We have made the following changes to the manuscript.

Results; lines 175-177 *Approximately 20000 different sets of model parameters were assessed using the Transitional Markov Chain Monte Carlo (TMCMC) method for each of the 32 datasets (Supplementary Section S.4, Tables S.1-S.5, Fig. S.9)*.

4. The paper is quite difficult to read as it is, it feels it was written to be read as Intro → Results → Discussion with Methods as an Appendix. This makes it quite hard to unpack the methods if reading in order. Id suggest restructure, or give the reader more signposts.

4. Reply: We thank the Reviewer for this comment. Indeed, the initial structure was less readable; therefore, we have now used the suggested structure.

Minor Comments:

5. Line 168-169 I don't see how Eq (2) is a signal production term... it looks like diffusion of chemotactic agent to me.

5. Reply: We thank the Reviewer for this comment. Indeed, our initial model included interaction terms between signals and cells; however, we performed a global sensitivity analysis and we found that his term did not contribute significantly to the model output and hence we discarded it. More information can be found in our previous work [5]. We have made the following changes to the manuscript.

Methods; lines 496-498 *In our previous analysis [5], we found that the signal production term in (2) was insensitive to the output; hence, we excluded it from the current model.*

2. I believe TMCMC is a special case of the widely used Sequential Monte Carlo. I would expect the authors to include references to the key literature in that space.

2. Reply: We included the following references related to the sequential Monte Carlo.

Bernardo, J. M., Bayarri, M. J., Berger, J. O., Dawid, A. P., Heckerman, D., Smith, A. F. M., West, M., Del Moral, P., Doucet, A., & Jasra, A. (2011). Sequential monte carlo for bayesian computation. Bayesian Statistics, 8, 1–34.

Chopin, N. (2002). A sequential particle filter method for static models. Biometrika, 89(3), 539–552.

Additional changes

Based on the Editor's comments we included an Author Summary section in lines 26-34 and a list of legends at the end of the manuscript.

References

1. Rørth, P. Collective Cell Migration. *Annual Review of Cell and Developmental Biology* **25,** 407–429. https://doi.org/10.1146/annurev.cellbio.042308.113231 (2009).

2. Carmona-Fontaine, C. *et al.* Contact inhibition of locomotion in vivo controls neural crest directional migration. *Nature* **456,** 957–961. ISSN: 1476-4687. https://doi.org/10.1038/nature07441 (2008).

3. Shellard, A. & Mayor, R. Rules of collective migration: from the wildebeest to the neural crest. *Philosophical Transactions of the Royal Society B: Biological Sciences* **375,** 20190387. https://royalsocietypublishing.org/doi/abs/10.1098/rstb.2019.0387 (2020).

4. Shellard, A., Szabó, A., Trepat, X. & Mayor, R. Supracellular contraction at the rear of neural crest cell groups drives collective chemotaxis. *Science* **362,** 339–343. https://www.science.org/doi/abs/10.1126/science.aau3301 (2018).

5. Dimitriou, N. M., Flores-Torres, S., Kinsella, J. M. & Mitsis, G. D. Quantifying the Morphology and Mechanisms of Cancer Progression in 3D in-vitro environments: Integrating Experiments and Multiscale Models. *IEEE Transactions on Biomedical Engineering.* ISSN: 15582531 (2022).

6. Lima, E. *et al.* Selection and validation of predictive models of radiation effects on tumor growth based on noninvasive imaging data. *Computer Methods in Applied Mechanics and Engineering* **327,** 277–305. ISSN: 0045-7825. https://www.sciencedirect.com/science/article/pii/S0045782517305893 (2017).

7. Lipková, J. *et al.* Personalized Radiotherapy Design for Glioblastoma: Integrating Mathematical Tumor Models, Multimodal Scans, and Bayesian Inference. *IEEE Transactions on Medical Imaging* **38,** 1875–1884. ISSN: 1558-254X (2019).

8. Liu, H., Lu, T., Kremers, G.-J., Seynhaeve, A. L. B. & ten Hagen, T. L. M. A microcarrier-based spheroid 3D invasion assay to monitor dynamic cell movement in extracellular matrix. *Biological Procedures Online* **22,** 3. ISSN: 1480-9222. https://doi.org/10.1186/s12575-019-0114-0 (2020).

9. Han, Y. L. *et al.* Cell contraction induces long-ranged stress stiffening in the extracellular matrix. *Proceedings of the National Academy of Sciences* **115,** 4075–4080. https://www.pnas.org/doi/abs/10.1073/pnas.1722619115 (2018).

10. Schrader, J. *et al.* Matrix stiffness modulates proliferation, chemotherapeutic response, and dormancy in hepatocellular carcinoma cells. *Hepatology* **53,** 1192–1205. https://aasldpubs.onlinelibrary.wiley.com/doi/abs/10.1002/hep.24108 (2011).

11. Loessner, D., Flegg, J. A., Byrne, H. M., Clements, J. A. & Hutmacher, D. W. Growth of confined cancer spheroids: a combined experimental and mathematical modelling approach. *Integrative Biology* **5,** 597–605. ISSN: 1757-9708. https://doi.org/10.1039/c3ib20252f (2013).

12. Jiang, T. *et al.* Engineering bioprintable alginate/gelatin composite hydrogels with tunable mechanical and cell adhesive properties to modulate tumor spheroid growth kinetics. *Biofabrication* **12,** 15024. ISSN: 17585090. https://doi.org/10.1088%7B%5C%%7D2F1758-5090%7B%5C%%7D2Fab3a5c (2020).

13. Boghaert, E. *et al.* Host epithelial geometry regulates breast cancer cell invasiveness. *Proceedings of the National Academy of Sciences* **109,** 19632 LP –19637. http://www.pnas.org/content/109/48/19632.abstract (2012).

14. Farrell, K., Oden, J. T. & Faghihi, D. A Bayesian framework for adaptive selection, calibration, and validation of coarse-grained models of atomistic systems. *Journal of Computational Physics* **295,** 189–208. ISSN: 0021-9991. https://www.sciencedirect.com/science/article/pii/S0021999115002430 (2015).

15. Lima, E. A. B. F., Oden, J. T., Hormuth, D. A., Yankeelov, T. E. & Almeida, R. C. Selection, calibration, and validation of models of tumor growth. *Mathematical Models and Methods in Applied Sciences* **26,** 2341–2368. https://doi.org/10.1142/S021820251650055X (2016).
