# Supplementary material for: Cancer cell sedimentation in 3D cultures reveals active migration regulated by self-generated gradients and adhesion sites
Source: PLoS Comput Biol. 2024 Jun 11;20(6):e1012112. doi: 10.1371/journal.pcbi.1012112 (PMC11195982; doi:10.1371/journal.pcbi.1012112)
Supplement: Attachment — Submitted filename: response_letter.pdf [file pcbi.1012112.s004.pdf]

# Response letter: PCOMPBIOL-D-23-00758R1

We would like to thank the editor and reviewers for the time spent on reviewing our manuscript, as well as for their thoughtful comments which have helped us improve the clarity and presentation of our findings. Below are our responses to the comments of the reviewers.

## Reviewer 1

List of minor comments:

1. Figure 3a: The large range of identified values for some of the parameters suggests that the mathematical model that the authors are using is not identifiable (that is, there may be more than one set of parameter values that can accurately capture the observed data). Can the authors comment on this possibility in the main text?

***1. Reply:*** We thank the Reviewer for this comment. Indeed, the increased range of the posterior distributions suggests identifiability issues. This is possibly due to sample misalignment under the microscope across different time-points, as well as small geometric differences of the 3D scaffold, which in-turn may have caused the model to fit in different parameter values. Also, we cannot the limited number of time-points that can also contribute to identifiability issues. To answer this, we have performed the following changes in the Discussion section.s

**Discussion Section B, lines 283-288** *Nevertheless, the increased range of the posterior distributions suggested identifiability issues, possibly owing to misaligned 3D culture samples across different time-points, small geometric differences across scaffolds, as well as limited number of time-points.*

2. Abstract: If there is space available, it may be helpful to introduce the importance of this problem in the abstract.

***2. Reply:*** We have made the following changes in the abstract.

**Abstract, lines 12-13** *Understanding this poorly examined phenomenon may allow us to design better protocols to prevent it, as well as provide insights into the mechanobiology of cancer development.*

3. Line 179: It would be helpful to list the biological interpretation of the parameter s, similar to the sentence previous to this.

***3 Reply:*** We included a description of $s$ and $D_f$. The following changes have been made.

**Results section F, lines 180-181** *... which represent the growth rate of the cells and the diffusivity of the chemotactic signals, respectively,...*

4. Line 232-233: Do the authors have results that confirm that fibroblasts upregulate their own protein / chemical secretion? Could another explanation be that cells adhere to the fibroblasts or are prevented from moving down due to overcrowding? Do the fibroblasts move from their original positions?

***4. Reply:*** Unfortunately, we cannot confirm that fibroblasts upregulate their own

protein, because we do not know the mechanism which stimulates their interaction with the cancer cells. We confirm that during the initial stages of the experiment, we observed both fibroblast network assembly, as well as their invasion in the ECM, which we believe was due to signalling with the cancer cells. We agree that their adhesion to the cancer cells is likely the reason that prevented downward migration. To make this more clear we made the following changes.

**Results section H, lines 238-240** *Thus, the adhesion of the fibroblasts to the cancer cells is likely the reason that prevented downward migration, which was preceded by the movement of the fibroblasts toward the cancer cells, likely due to signaling.*

5. Line 256: Can the authors cite a reference for this fact about matrigels?

***5. Reply:*** We provide the following reference that supports this statement. **Zaman, M. H., Trapani, L. M., Sieminski, A. L., MacKellar, D., Gong, H., Kamm, R. D., Wells, A., Lauffenburger, D. A., & Matsudaira, P. (2006). Migration of tumor cells in 3D matrices is governed by matrix stiffness along with cell-matrix adhesion and proteolysis. Proceedings of the National Academy of Sciences, 103(29), 10889–10894**. In general, we expect that crosslinked hydrogels present no fiber alignment, unless there is an external stimulus such as elongation forces that can cause some level of fiber alignment.

6. Line 290: Do the authors mean in this sentence that contact of inhibition may also be present, based on the expression profiles in the treated and control samples?

***6. Reply:*** Yes, it is possible that contact inhibition is a contributor of migration.

7. References 13, 41, 53, 91, 94: The link / DOI given in this section does not work.

***7. Reply:*** We corrected the corrupted links.

8. Supplementary Figure S.14: It may be helpful to state in the caption what parameter values correspond to the low diffusion / high advection and high diffusion / low advection states.

***8. Reply:*** We made the following changes.

**Supplementary Figure S.14, caption:** *... one with low advection* ($0.0005 \pm 0.0004$ $mm^2/day$) *and high diffusion* ($0.0076 \pm 0.0003$ $mm^2/day$) *constants, and one with high advection* ($0.0052 \pm 0.0002$ $mm^2/day$) *and low diffusion* ($0.00186 \pm 0.00009$ $mm^2/day$)*...*

9. Lines 410-411: Did the authors examine the stiffness of their gels, and whether there actually is a durotactic gradient?

***9. Reply:*** Thank you for your comment. Since the size of the Matrigel scaffold was small we were not able to test for the presence of gradients that may have induced durotactic motion. We mention the possible presence of durotaxis in line 253 of the Discusion section A.

## Reviewer 2

I recommend the manuscript for publication as it is.

***Reply:*** Thank you.

## Reviewer 3

I thank the authors considered responses to my initial comments. I have no other concerns, perhaps a small addition to the manuscript about why no model selection was performed would be useful to a reader (the authors' response to my comment 2 should be reflected somewhere in the main manuscript). Computational limitations are perfectly legitimate reasons.

***Reply:*** Thank you. We have made the following changes.

**Discussion section B, lines 286-288** *The examination of different models could allow us to find a model with better balance between complexity and fitness, however model selection was not performed due to limited computational power.*
